# Understanding Economic Decision-Making in Digital Therapeutics Development: Qualitative Approach

**DOI:** 10.2196/79746

**Published:** 2025-09-16

**Authors:** Yoann Sapanel, L Martin Cloutier, Alec Morton, Sapphire Lin, Gyula Seres, Dean Ho

**Affiliations:** 1 The Institute for Digital Medicine (WisDM), Yong Loo Lin School of Medicine National University of Singapore Singapore Singapore; 2 The N.1 Institute for Health National University of Singapore Singapore Singapore; 3 Singapore’s Health District @ Queenstown, Yong Loo Lin School of Medicine National University of Singapore Singapore Singapore; 4 Department of Analytics, Operations, and Information Technologies University of Quebec at Montreal Montreal, QC Canada; 5 Saw Swee Hock School of Public Health National University of Singapore Singapore Singapore; 6 Department of Biomedical Engineering, College of Design and Engineering National University of Singapore Singapore Singapore; 7 Department of Pharmacology, Yong Loo Lin School of Medicine National University of Singapore Singapore Singapore

**Keywords:** digital health, digital therapeutics, economic evaluation, cost-effectiveness, qualitative study, decision-making, critical realism, decision theory, system dynamics

## Abstract

**Background:**

Digital therapeutics (DTx) represent a transformative shift in health care delivery, offering software-driven, evidence-based therapeutic interventions. Despite their potential, adoption remains low across health care systems, partly due to insufficient economic evidence. Significant knowledge gaps persist regarding stakeholders’ approaches to economic decisions in DTx development, with prior studies also indicating limited consideration of economic factors in early DTx development stages, particularly from researchers.

**Objective:**

This study investigates how researchers approach decision-making regarding factors that influence the economic impact of DTx during technological development and clinical validation phases, examining the underlying mechanisms and contextual conditions that shape these processes.

**Methods:**

Using a critical realism philosophical stance, 17 semistructured interviews were conducted with researchers involved in DTx development, including research engineers (n=5), health systems and social science researchers (n=6), clinician-researchers (n=4), and practitioner-researchers (n=2). The research approach combined deductive and inductive coding, followed by abductive and retroductive inference processes to identify generative mechanisms underlying observed decision-making patterns. Qualitative system dynamics modeling was applied to visualize causal loop relationships through triangulated data sources.

**Results:**

Three interrelated generative mechanisms were identified that shape researchers’ decision-making regarding economic considerations: (1) the professional norms, operating through reinforcing loops that systematically prioritize clinical validation while marginalizing economic considerations; (2) the researcher experience, revealing how professional training and limited economic literacy create cognitive biases that obscure economic factors; and (3) the DTx adoption uncertainties, demonstrating how implementation concerns influence development decisions through both reinforcing and balancing feedback loop dynamics. These mechanisms explain why, despite growing recognition of the importance of economic evidence, economic considerations remain peripheral in researchers’ decision frameworks.

**Conclusions:**

This study reveals complex interactions between institutional structures, intrapersonal factors, and implementation uncertainties that systematically deprioritize economic considerations in DTx development. The identified mechanisms provide valuable intervention points for strengthening the development process toward a more comprehensive assessment of clinical, technical, and economic value throughout the DTx lifecycle to ultimately enhance their adoption in health care systems.

## Introduction

### Background

Digital therapeutics (DTx) represent a transformative shift in health care delivery, offering software-driven, evidence-based therapeutic interventions that prevent, manage, and treat a diverse spectrum of medical conditions [1]. These digital solutions, ranging from mobile apps to virtual reality environments, are designed to improve patient outcomes through evidence-based therapeutic interventions. The promise of DTx lies in its ability to provide accessible, scalable, and personalized interventions. For instance, mobile apps can deliver cognitive behavioral therapy for mental health conditions, while virtual reality environments may assist in pain management or rehabilitation [2,3]. This emerging class of medicine can potentially reach underserved populations, provide continuous support between clinical visits, and generate real-time data for health care providers to optimize treatment plans.

In the context of global population aging and the concomitant rise in chronic and mental health conditions, DTx offer the opportunity to improve patient outcomes while potentially mitigating the escalating costs associated with traditional health care delivery. However, notwithstanding their potential, the literature is unanimous: the adoption of these therapeutics remains low across countries and health care systems, with limited integration of DTx into access and care pathways [4-8].

The path from concept to market implementation is marked by unique levels of complexity, which differentiate DTx from conventional pharmaceutical drugs and medical devices. These complexities stem from the rapid pace of technological evolution and the intersection of software development with clinical validation requirements. Additional challenges include the “regulatory gap” in approval and reimbursement processes across countries and the numerous decision-making processes involving stakeholders from multiple disciplines [8-10].

With health care systems under pressure to “do more with less,” robust clinical and economic evidence is critical for DTx to be considered for regulatory and reimbursement decisions [6,10-13]. Recent studies also underscore that economic factors related to DTx interventions—such as costs, funding, and resources—have been identified as both barriers and facilitators to adoption and use [14]. However, relatively little is known about the capacity of DTx to provide economic value in care [15-17].

### Stakeholders’ Role in Driving DTx Economic Impact

Recent research has extensively examined decision-making in health care innovation [18,19], yet significant knowledge gaps persist regarding stakeholders’ approaches to economic decisions in DTx development—a process that encompasses both the technological development phase and subsequent clinical validation through interventional trials. Although the literature offers valuable insights into clinical decision-making processes [11,20] and technical development frameworks [10,21], the intersection of economic considerations with these domains remains understudied.

A recent study exploring stakeholder roles in driving DTx economic impact revealed a significant pattern: researchers consistently placed higher emphasis on technical validation while giving less consideration to economic and implementation factors compared to other stakeholders [22]. This disparity raises important questions about the underlying causes of researchers’ apparent deprioritization of these considerations.

While researchers play a crucial role in establishing the scientific foundation and clinical efficacy of DTx solutions [7], their limited consideration of economic factors could potentially impact downstream commercial viability and health care system integration. Despite economic evidence being critical for adoption, there remains a limited understanding of how stakeholders approach economic decisions in DTx development, particularly how researchers’ early-stage decision-making impacts downstream value creation.

Thus, the following research question is raised: What are the underlying mechanisms and contextual conditions that shape researchers’ decision-making processes regarding economic factors perceived to influence a DTx economic value in its technological development and clinical validation, and how do these manifest in practices?

### Objectives

This study used a qualitative, exploratory methodology to investigate how researchers perceive and evaluate factors that influence the economic value of DTx. Specifically, it aims to uncover the underlying mechanisms that drive these considerations and examine how they are operationalized during DTx development.

This paper seeks to reveal why they consistently give less consideration to economic factors compared to other DTx stakeholders, and how these decisions operate within complex systems-based relationships. This understanding could ultimately lead to more effective DTx development processes that better integrate economic considerations, potentially improving the adoption and use rates of these promising health care interventions.

## Methods

### Study Design

This study is grounded in critical realism (CR) as its ontological and epistemological foundation, enabling the analysis to move beyond observable patterns in researchers’ decision-making practices (empirical domain) to identify underlying generative mechanisms (real domain) that produce such observable patterns [23]. These mechanisms would have been invisible through purely empirical approaches [24].

Building on this philosophical foundation, decision theory (DT) provides a robust theoretical and analytical framework for examining complex choice-making processes under uncertainty—a central challenge in health care innovation [25]. This study uses both normative and descriptive components of DT [26]. Expected utility theory (EUT) establishes benchmarks for optimal decision-making, while behavioral decision theory (BDT) explains deviations through cognitive biases and bounded rationality that characterize real-world health care innovation.

Systems thinking, implemented through qualitative system dynamics (QSD) methodology, provides structure and syntax to visualize and map how these decision-making processes are interrelated in practice. Causal loop diagrams (CLDs) reveal the interconnected variables, feedback loops, and causal relationship structures within each generative mechanism to enable the identification of potential intervention or leverage points [27,28]. The integrated theoretical framework guiding this study and informing the methodological approach is detailed in Multimedia Appendix 1 [23-51].

### Participant Selection and Recruitment

The decision-making processes of 17 researchers were investigated, including 5 research engineers, 6 health systems and social science researchers, 4 clinician-researchers, and 2 practitioner-researchers. Table 1 summarizes participant characteristics, with comprehensive details provided in Multimedia Appendix 2. A nonprobabilistic purposeful sampling procedure was used to identify and select participants based on their relevant experience within specific contexts [52], to facilitate comparative analysis, and to meet the following inclusion criteria: (1) a minimum of 5 years of experience in DTx development or clinical validation, (2) current involvement in the technological development or clinical validation of a DTx, and (3) fluency in English or French. Initial recruitment occurred through the research team’s professional network, with participants encouraged to refer qualified colleagues through snowball sampling.

This purposeful sampling approach used an iterative selection process that enabled responsive participant recruitment based on emerging analytical insights [53]. Data collection used a “two-phase” recruitment strategy over 6 months, designed to capture diverse perspectives from “information-rich informants” while maintaining methodological rigor consistent with purposeful sampling principles [52]. This iterative process allowed the second phase of interviews to explore adjacent concepts and emerging codes identified during the initial phase, ensuring theoretical saturation and depth of understanding.

**Table 1:** Demographic data summary of study participants.

Number			Pseudonym^a^		Field of practice		Setting (country)
**Research engineers**							
	P1	Emma		Biomedical engineering		Public academic institution (Singapore)	
	P2	Eva		Biomedical engineering		Public academic institution (United States)	
	P3	Ezra		Biomedical engineering		Public academic institution (Singapore)	
	P4	Eisa		Biomedical engineering		Public academic institution (Singapore)	
	P5	Elisabeth		Neuroengineering		Private health care organization (Switzerland)	
**Health systems and social science researchers**							
	P6	Sasha		Behavioral sciences		Public academic institution (Singapore)	
	P7	Sara		Health care management		Public academic institution (Germany)	
	P8	Senna		Health policy		Public academic institution (United Kingdom)	
	P9	Shiva		Health policy		Public academic institution (Singapore)	
	P10	Surya		Health economics		Public academic institution (United Kingdom)	
	P11	Santana		Health economics		Public academic institution (Germany)	
**Clinician-researchers**							
	P12	Christoph		Neuropsychology		Private health care provider (Australia)	
	P13	Cheah		Ophthalmology		Public health care provider (Singapore)	
	P14	Chevannah		Cardiology		Private health care organization (Germany)	
	P15	Camirah		Nursing		Public health care provider (Singapore)	
**Practitioner-researchers**							
	P16	Priscillah		Technology implementation		Public health care provider (Canada)	
	P17	Priyah		Technology implementation		Public health care provider (Singapore)	

^a^The pseudonyms are designed to convey key information about each participant. The first letter indicates their role: “E” for research-engineers, “S” for health systems and social science researchers, “C” for clinician-researchers, and “P” for practitioner-researchers. The last letter denotes their current work setting: “A” for academic organization, and “H” for health care providers or organizations (either private or public). The gender of the pseudonym corresponds to the participant’s sex. For instance, the pseudonym “Christoph” (P12) signifies a male clinician-researcher working in a health care organization or provider.

### Ethical Considerations

The study was approved by the Ethics Boards of the National University of Singapore (NUS-IRB-2023-399) and the University of Quebec at Montreal (UQAM-CIEREH-2024-6028). Written informed consent was obtained from participants to engage in either in-person or web-based interviews. No compensation or incentive was provided. All interviews were audio-recorded and transcribed in their entirety. Interview data were securely stored on protected servers with access restricted to the research team. To ensure participant privacy and confidentiality, data were labeled with participant codes, and a separate secure list of code-to-name matchups was maintained. The study was conducted according to the COREQ (Consolidated Criteria for Reporting Qualitative Research) guidelines (Multimedia Appendix 3 [54,55]) [56].

### Data Collection

Semistructured interviews were conducted between April and September 2024, either in-person or via videoconferencing, lasting 30-60 minutes. This interview format balanced an exploration of existing literature on researchers’ decision-making processes while allowing the emergence of novel insights. The interview guide (Multimedia Appendix 4) covered topics including: (1) the participants’ roles as DTx researchers, (2) their priorities and key considerations throughout the DTx lifecycle, and (3) their day-to-day prioritization and decision-making processes.

The interview questions evolved iteratively both within and between interviews throughout the data collection process. Early interviews revealed the importance of implementation considerations, leading to additional questions about how researchers anticipated and addressed implementation challenges. Similarly, emerging themes around professional training prompted new questions about how researchers’ educational backgrounds influenced their approach to economic considerations.

Detailed field notes were taken during and immediately after each interview to capture nonverbal cues and emerging insights. Data collection continued until theoretical saturation, when additional interviews yielded no substantial new themes or insights and resulted in “little or no change to the codebook” [57,58].

### Data Analysis

#### Overview

The data analysis followed a structured three-step analytical framework grounded in CR principles, as developed by Fletcher [54]. This approach progressed through coding and identification of demi-regularities, abduction, and retroduction—with each phase building upon the previous one. Throughout each analytical step, biweekly member checking with the research team and regular peer debriefing sessions were conducted to minimize potential bias and ensure analytical rigor. Multimedia Appendix 5 [30,54,55,59] outlines these key analytical steps, which uncovered the underlying mechanisms driving decision-making in DTx development.

#### Step 1: Coding and Identification of Demi-Regularities

A “deductive yet flexible” [54] coding process was implemented that synthesized theoretical foundations and existing literature, while also capturing CR’s stratified ontology to develop the initial coding framework. This approach began with the creation of a theoretically informed codebook derived from multiple complementary sources.

First, key concepts from DT were extracted, specifically BDT and EUT, to develop foundational codes (Multimedia Appendix 1 [23-51]). These included “Bias > Availability heuristic” (capturing researchers’ tendency to overestimate the likelihood of events based on recall ease) and “Probability > Probability calculations” (documenting objective and subjective probability assessments in DTx development decision-making). Health technology assessment principles from EUT were further incorporated, generating codes, such as “Economic Considerations > Economic evaluation methods” to document approaches for assessing DTx cost-effectiveness, including cost-benefit analysis, cost-effectiveness analysis, and cost-utility analysis.

Second, the theoretical framework was enriched by integrating findings from recent DTx development literature [4,6,8,10,17,22,60,61]. For example, “Economic Considerations > Funding sources” captured available financial resources and funding options that van Kessel et al [61] described as “vital” during and postresearch DTx phases. Similarly, “Economic Considerations > Implementation related” documented researchers’ awareness of practical deployment considerations, such as clinical workflow integration and provider training needs, which Cirkel et al [4] identified as primary barriers to DTx adoption.

To ensure philosophical alignment with the research approach, codes derived from CR tenets were integrated. These included “Knowledge Integration > Patient feedback integration” to document attempts to incorporate patient perspectives into the DTx development process, and “DTx Development > Patterns” to identify recurring approaches in development processes and decision-making.

Most codes appeared across multiple sources: theoretical literature, empirical research, and CR principles. For instance, “Bias > Overconfidence,” addressing the tendency to overestimate one’s abilities and prediction accuracy, appears both in CR as a cognitive constraint and in BDT as a fundamental decision-making bias.

**Figure 1:**
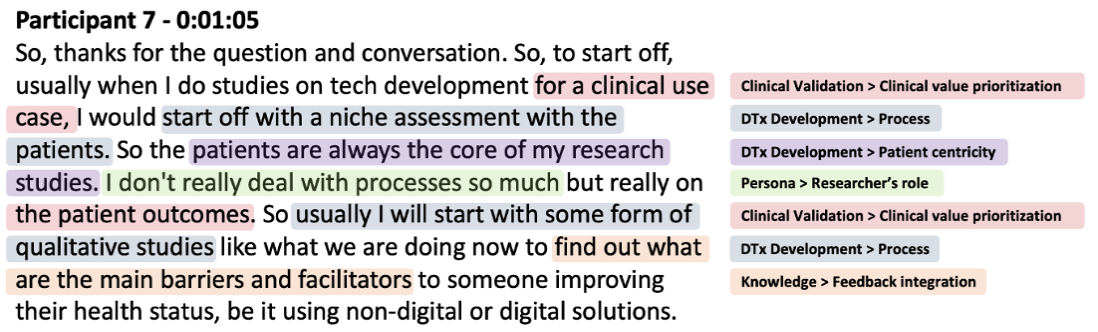
Sample coded participant interview transcript demonstrating thematic analysis with color-coded categories.

The initial codebook comprised 58 theoretically derived codes (Multimedia Appendix 6). This framework was applied to transcripts using ATLAS.ti software (ATLAS.ti Scientific Software Development GmbH). Throughout this iterative coding process, a comprehensive audit trail was maintained to document all coding decisions, rationale for code modifications, and the systematic evolution of the codebook from its initial 58 codes to the final framework of 78 codes. These memos served to support the validity of every stage of data collection and analysis [62]. Figure 1 illustrates a representative sample of the coded transcript analysis.

In alignment with CR principles, while the deductive approach provided theoretical foundations, an inductive process was incorporated to ensure an authentic representation of participants’ perspectives [63]. To systematically analyze the data, Saldaña’s [55] cycles of coding methodology were applied throughout the coding process (Multimedia Appendix 7 [54,55]).

Codes were integrated into a coherent analytical narrative that connected empirical observations with theoretical constructs. This process facilitated the identification of tendencies in empirical data—what CR practitioners term “demi-regularities”—by searching for recurring patterns in participants’ experiences and perspectives within the developed codes [54].

Through subsequent cycles of axial coding, enhanced by ATLAS.ti software’s visualization tools—including word clouds and treemaps where code size dynamically reflects app frequency—commonly coded elements and their relationships were systematically explored and mapped. This analytical approach enabled the establishment of connections between second-order categories, progressively distilling the data into eight third-order subcategories (Multimedia Appendix 7 [54,55]). These were ultimately consolidated into three overarching core themes discussed in the Results section: (1) the centrality of clinical evidence, (2) the importance of implementation considerations, and (3) the notable absence of economic value consideration.

#### Step 2: Abduction

Following CR principles, the analysis proceeded through complementary abductive (step 2) and retroductive processes (step 3) [54]. Abductive reasoning is a process of forming explanatory hypotheses for less-understood phenomena [64]. Dunne and Dougherty [64] defined abduction or retroduction as a form of “reasoning that generates and evaluates hypotheses in order to make sense of puzzling facts.” Sætre and Van de Ven [59] highlighted that abduction “begins with observing and confirming an anomaly, and generating and evaluating hunches that may explain the anomaly, for subsequent deductive constructing and inductive testing.” Critically, abductive reasoning does not produce definitive answers but suggests plausible explanations that warrant further investigation.

Sætre and Van de Ven’s [59] four-step approach was adopted for the abductive reasoning process, which they note, “may overlap, iterate, and unfold in stochastic ways over time.” The first step involves observing anomalies or puzzling facts in the empirical data that might not align with initial assumptions or existing models and thus remain unexplained. The second step confirms that such anomalies exist and are grounded in evidence, defining what is known or unknown about them. The third step involves formulating hypotheses or generating hunches that may provide plausible explanations for the anomalies. The final step consists of evaluating these hunches and alternative explanations.

Through this process, the iterative movement between empirical data and theoretical literature identified potential puzzling facts and initial patterns in researchers’ decision-making processes. The Results section reconceptualizes and redescribes these puzzling facts and provides plausible explanations for these observations.

#### Step 3: Retroduction

Through retroduction, the final stage of CR analysis, the causal mechanisms and conditions underlying the observed patterns in researchers’ decision-making were investigated [29,65]. Retroduction involves reasoning from observed demi-regularities to the underlying structures and causal powers that make them possible [23]. These mechanisms, defined by Bhaskar [23] as the “causal structures that trigger events or outcomes,” represent the layer of the “actual.” This investigation revealed deeper causal powers and structures producing observable phenomena, moving beyond surface-level correlations or patterns. The analysis specifically examined interrelated mechanisms that either stimulate or constrain the consideration of economic factors in DTx development.

Following Danermark et al’s [30] framework, three fundamental questions were explored: How were these patterns in researchers’ decision-making possible? What properties were essential to their existence? What causal mechanisms drove them? This investigation involved iterative analysis of data from steps 1 and 2.

To illustrate the retroduction process, we examined the “absence of economic value consideration” demi-regularity—a robust observable pattern identified across 17 researchers from diverse backgrounds and contexts, with only one exception (Cheah, P13). Through retroduction, the following questions were raised: What underlying structures and causal powers make this absence not merely possible, but systematically reproduced across different researchers, institutions, and DTx projects? Why is this pattern so robust that it persists even when researchers acknowledge the importance of economic factors for implementation success?

The first alternative explanation examined was the knowledge deficit hypothesis, which predicted that economic training would eliminate the absence pattern. However, this was found to be insufficient as the sole explanation. Despite their expertise as health economists, Surya (P10) and Santana (P11) were found to operate within institutional frameworks that marginalized economic considerations in DTx projects. The second alternative explanation tested was the individual preference hypothesis, which predicted that some researchers naturally gravitate toward clinical or technical domains, while others prefer economic analysis. This explanation was also found to be insufficient, given the pattern’s consistency across personality types, career stages, and disciplinary backgrounds. Even business-oriented researchers like Priscillah (P16) exhibited the same pattern of marginalizing economic considerations. The third alternative explanation was the rational temporal sequencing hypothesis, which predicted that researchers deliberately postpone economic considerations because early-stage technical uncertainty makes economic evaluation premature. This was falsified by data showing that strategic temporal decisions about when to introduce economic factors were not being made. Instead, satisficing behavior was exhibited, with researchers defaulting to familiar domains rather than systematically planning economic integration points.

This systematic explanation testing process revealed that no single-level explanation could account for the pattern’s robustness. For each demi-regularity, such systematic and iterative questioning processes were implemented. Working backward from observed effects to theorize underlying causes was then undertaken, with mechanism propositions being iteratively tested against the data.

This process ultimately led to the identification of three primary generative mechanisms that best explain the observed patterns in researchers’ decision-making practices: (1) the professional norms, (2) the researcher experience, and (3) the adoption uncertainties. These mechanisms are discussed in the Results section, with illustrative quotes in Multimedia Appendix 8.

Drawing on the methodological approaches of Lawani [66], Stohr et al [31], and Nguyen et al [67], QSD was applied to enhance understanding, visualize, and synthesize to articulate how these mechanisms and their components manifest and influence each other through causal relationships via a CLD. QSD and SD modeling have been used extensively in health care to better understand complex behaviors and interconnected challenges in health care technology development [68-71].

To conceptualize these mechanisms and elucidate their interdependencies through a CLD, an iterative three-step process was implemented to: (1) identify influencing variables, (2) map relationships and assign polarity, and (3) validate and refine the model. Throughout each phase, three distinct categories of unit triangulation were incorporated [72], comprising multiple sources of data, iterative triangulation with existing literature, and investigator triangulation, as detailed in Multimedia Appendix 9 [22,72-75].

These triangulation categories were used to serve CR aims in developing deeper explanatory accounts while acknowledging knowledge limitations, ultimately capturing the complexity involved in the system of interrelated variables shaping researchers’ decision-making and their considerations of economic aspects. This process facilitated the refinement and validation of identified generative mechanisms while visually mapping the cause-and-effect relationships underlying DTx decision-making processes [31]. Ultimately, this triangulation approach aimed to achieve a higher degree of internal and external validity, strengthening confidence in the findings [72].

### Critical Realism’s Contribution

CR compelled examination of why these identified demi-regularities are structurally reproduced across contexts, rather than simply categorizing them as an emergent theme (such as “lack of economic consideration”) derived from participant accounts. This approach revealed that the pattern resulted not merely from individual researcher choices, but from underlying mechanisms operating independently of conscious awareness, thereby providing explanatory depth beyond descriptive categorization [23].

Notably, Cheah (P13) represented an exception to this pattern. CR required that the structural conditions enabling his distinctive approach (exposure to economic concepts during his specialization training) be identified, which strengthened understanding of the underlying mechanisms [76].

CR thus revealed a fundamental contradiction embedded within the DTx development process: while researchers demonstrated awareness of economic importance at the empirical level, their practices contradicted this awareness at the actual level because deeper structural constraints at the real level systematically limited their decision-making options. As it will be discussed in the next section, this three-tiered analysis revealed how institutional norms, professional training, and implementation uncertainties interact to shape research practices in ways that individual awareness or intention alone cannot overcome. The identification of these generative mechanisms provided the foundation for developing targeted intervention strategies that address root causes rather than surface symptoms, ultimately enabling more integrated approaches to DTx development that better balance clinical efficacy with economic sustainability.

### Methodological Safeguards

Multiple methodological safeguards were implemented to ensure the validity, reliability, and trustworthiness of the study findings.

Internal reliability was ensured through several analytical procedures [62]: (1) biweekly peer debriefing sessions were conducted between research team members to review coded transcripts and establish consensus on emerging themes; (2) a comprehensive audit trail was maintained to document all coding decisions, modifications, and the systematic evolution of the codebook; and (3) iterative retroduction process was used to test hypothesized generative mechanisms against empirical patterns.

External validity was established through triangulation approaches [77], whereby the analysis was grounded in established theoretical frameworks (CR, DT, and systems thinking) and findings were systematically compared with existing literature. The theoretical soundness of emerging CLDs was validated by three external DTx experts meeting study inclusion criteria at different analytical intervals, providing preliminary evidence of pattern recognition and transferability within the field (Multimedia Appendix 3 [54,55]).

These combined methodological approaches collectively establish the credibility and transferability of findings to similar research contexts.

## Results

### Overview

Content analysis at the first step, “Coding and Identification of Demi-regularities,” revealed three core themes: (1) the centrality of clinical evidence in the DTx development processes, (2) the crucial role of implementation factors in development decisions, and (3) the consistent absence of economic value considerations in early development stages (Multimedia Appendix 10 [17,22,59,78-81]).

The subsequent abductive analysis (step 2) revealed key puzzling facts in DTx researchers’ decision-making processes, suggesting a complex interplay between institutional logics, individual expertise boundaries, and conceptual frameworks (Multimedia Appendix 10 [17,22,59,78-81]).

Three primary generative mechanisms were then reintroduced (step 3) to explain how researchers’ approach to DTx development and their consideration of economic factors: (1) the professional norms, (2) the researcher experience, and (3) the adoption uncertainties.

Each mechanism is examined individually below through CLDs, identifying respective variables (the measurable factors that can change within each mechanism) and their primary balancing (B) or reinforcing (R) loops. Primary loops are feedback loops that operate entirely within a single mechanism. These loops contain strong causal relationships between closely related variables and represent the essential dynamics within a distinct part of the system. Primary loops typically demonstrate immediate and direct impacts on key system variables. Secondary loops, by contrast, connect across different mechanisms within the system, creating bridges between primary loops. These loops often contain at least one weak causal link and exert less direct influence than primary loops. Secondary loops reveal how effects propagate throughout the entire system, showing cross-mechanism interactions that create more complex emergent behaviors than would be visible from primary loops alone.

**Figure 2:**
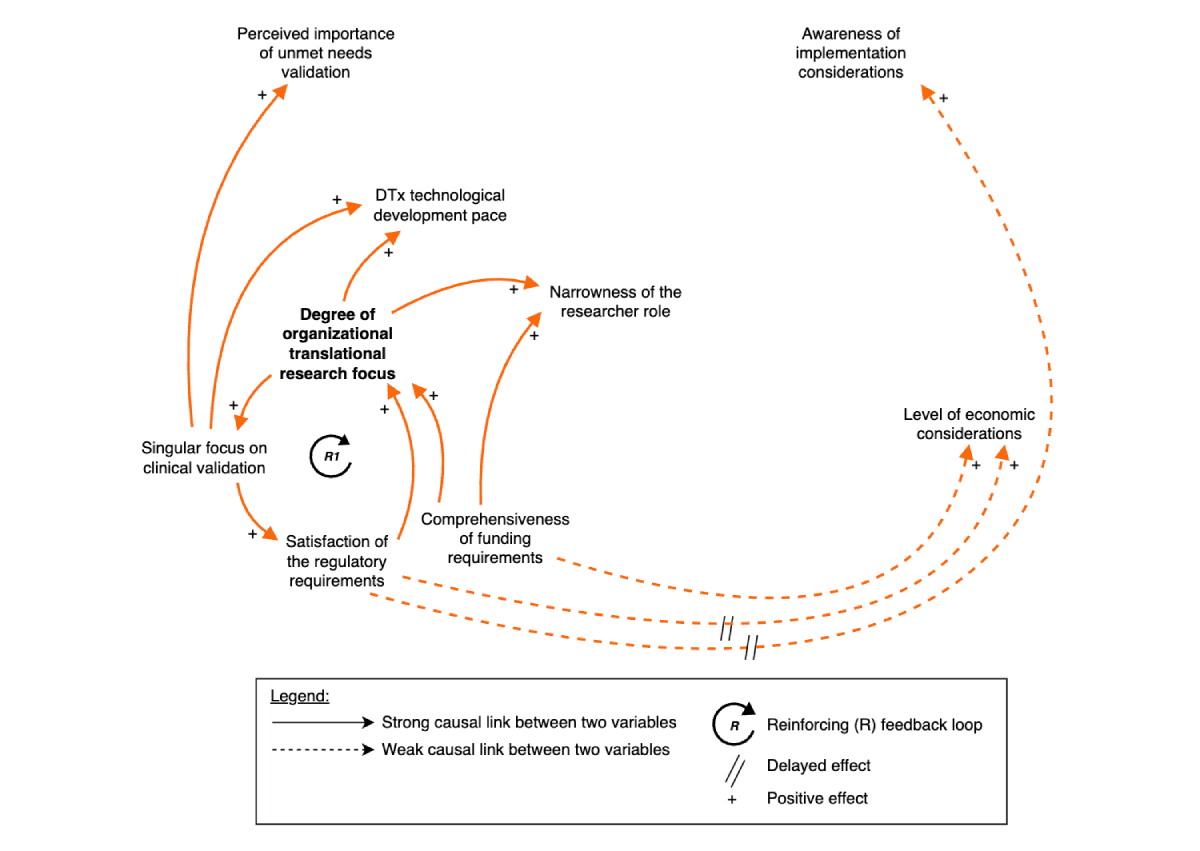
The causal loop diagram showing the professional norms mechanism and its manifestations shaping economic considerations in DTx development. This figure introduces the professional norms mechanism that illustrates how health care norms, institutional settings, and regulatory requirements, which are reinforced by funding requirements, create a reinforcing loop (R1: clinical value loop) that prioritizes clinical validation while marginalizing economic considerations in DTx development (emerging connection indicated in dashed line). DTx: digital therapeutics.

**Figure 3:**
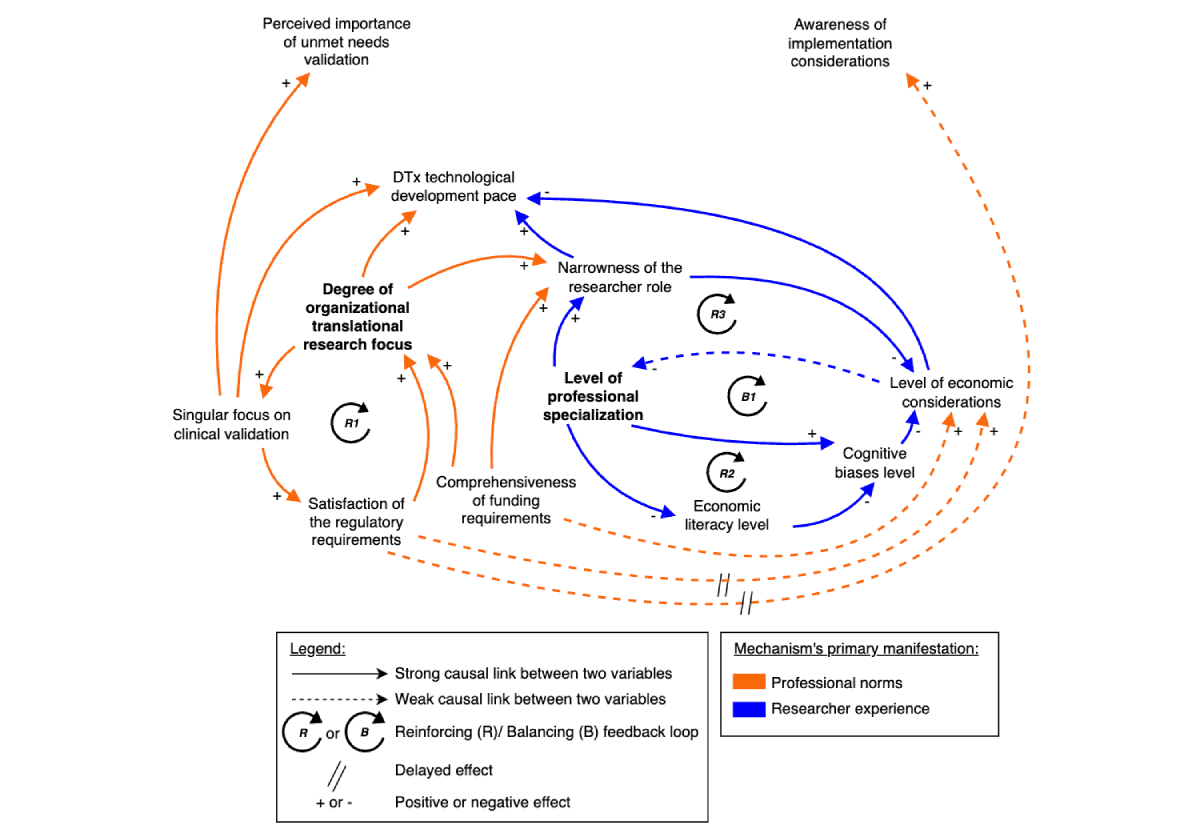
The causal loop diagram showing the researcher experience mechanism and its manifestations shaping economic considerations in DTx development. This figure introduces the researcher experience mechanism that demonstrates how researchers’ professional background, training, and cognitive biases create two reinforcing loops (R2: literacy loop and R3: role boundary loop) and one balancing loop (B1: bias loop) that systematically channel focus toward familiar domains and away from economic considerations. DTx: digital therapeutics.

**Figure 4:**
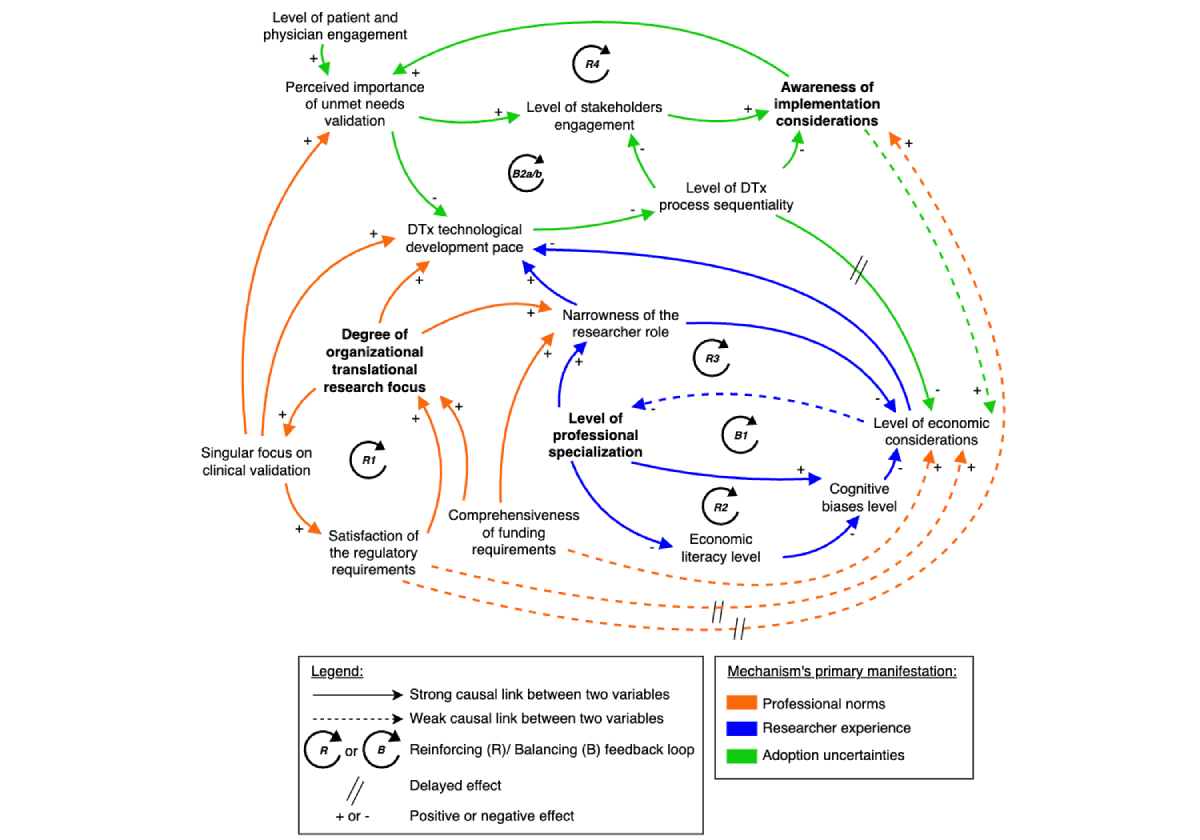
An integrated model combining the three identified mechanisms into a comprehensive system, illustrating the mechanisms’ manifestations and cross-interactions that shape economic considerations in DTx development. This figure introduces the adoption uncertainties mechanism that illustrates how implementation challenges create dynamic interactions between two balancing loops (B2a: implementation-driven development and B2b: stakeholder-driven development) and one reinforcing loop (R4: collaboration). This mechanism reveals how researchers’ awareness of adoption challenges drives increased focus on clinical needs, while stakeholder engagement simultaneously influences development approaches. Additionally, the figure presents an integrated model that combines all three generative mechanisms (professional norms, researcher experience, and adoption uncertainties) with their respective primary loops, demonstrating how institutional forces, researcher intrapersonal factors, and implementation uncertainties collectively influence economic considerations throughout the DTx development lifecycle. DTx: digital therapeutics.

Theoretical propositions are developed for each mechanism to explain how causal relationships influence researchers’ decision frameworks. It should be noted that the term “causal” as used in this qualitative study does not imply statistical causation as established through experimental design or quantitative methods. Rather, it refers to theorized causal mechanisms and relationships that emerge from participants’ lived experiences and narratives. This understanding of causality aligns with critical realist approaches that seek to identify underlying generative mechanisms while acknowledging the contextual, complex, and often nonlinear nature of social phenomena [54]. In addition, weak versus strong causal links, or the “importance” of a relationship, is evaluated by both the frequency and intensity of identified connections. Causal links were categorized as “weak” when mentioned by only a minority of stakeholders (ie, 1-2 researchers) or described by respondents using qualifying language, such as having “somewhat” of an influence or being “emerging,” “nascent,” or “weak” in nature. Conversely, strong causal links met two critical criteria: broader stakeholder recognition (acknowledged by at least 3 researchers) and substantial supporting evidence (frequently emphasized in interview transcripts as having a direct, significant impact on core system behavior). Building on each mechanism’s CLD (Figures 2-4), an integrated model combining the three mechanisms into a comprehensive system is presented in Figure 4.

### The Professional Norms Mechanism

#### Overview

The first mechanism, professional norms, refers to how embedded health care norms and practices fundamentally influence the development trajectories of DTx. This mechanism operates through a reinforcing loop (R1) alongside an exogenous variable—the comprehensiveness of funding requirements. These professional norms create self-perpetuating patterns that systematically prioritize clinical validation while marginalizing other forms of complementary evidence or value dimensions.

Sara’s (P7) reflections provide initial insight into how this mechanism operates in practice:

DTx products must first demonstrate robust clinical benefits before gaining market access, and undergo rigorous evaluation to determine whether they are clinically efficacious to warrant market authorization. Pricing discussions with manufacturers occur only after this clinical validation has been established. [...] In Germany, manufacturers can pursue one of two pathways: either demonstrating their DTx clinical value or proving process or structural improvements for the health system. However, in practice, approximately 95% or more utilize the clinical value pathway rather than focus on process improvements.

This clinical prioritization is further reinforced by funding structures. Camirah (P15) observed that grant and funding requirements have historically focused exclusively on clinical impact, with only recent initiatives beginning to incorporate economic dimensions into the grant review process.

The professional norms mechanism functions through an interconnected set of causal relationships that collectively prioritize clinical evidence while marginalizing economic considerations (Figure 2). This pattern, while unsurprising within health care contexts, significantly constrains the development process and highlights the persistent tension between rational decision-making frameworks and behavioral influences that drive researcher choices.

#### Reinforcing Loop 1 (R1): Clinical Value Loop

In R1, the degree of organizational translational research focus emerges as a primary influence, bolded in Figure 2, to express the centrality of this node of the causal model. The findings reveal an important aspect that influences researchers’ decision frameworks: the translational nature of the research institution, as hypothesized during step 2 of abduction (Multimedia Appendix 10 [17,22,59,78-81]).

Researchers in our sample worked across institutes. Their roles cover varying orientations, from basic scientific and technological development to clinically focused or translational research bridging academia and health care practice. These institutional visions exist along a continuum from basic science to translational approaches oriented toward commercialization. Such positions influence both institutional and individual research agendas, creating predetermined key focus areas and performance metrics for researchers. This effect is exemplified in how Ezra (P3) described the relationship between his lab’s vision and their output:

The aim of our lab is to do something that could potentially impact society. We consider multiple aspects, such as implementation or translation. We’re not just doing research for the sake of publishing, nor are we focused solely on basic science. We want to do something impactful that could potentially be translated into real-life benefits for the general public.

Team composition and multidisciplinary expertise within research organizations directly impacted by the translational nature of these organizations shape researchers’ mindsets and priorities. Engineer-researcher Eva’s (P2) experience working alongside behavioral scientists illustrates this dynamic:

Researchers like me, we are too focused on numbers, like how effective is the solution, and our expertise domain. When I joined [Bioengineering Research Institute Name], I was surprised about the behavioral component. I didn't appreciate it at first, but now I see its importance. It opened my mind and perspective, especially for improving DTx usability and engagement.

This excerpt demonstrates how diversifying team composition can potentially disrupt R1 by introducing alternative perspectives, although the data suggest such disruptions rarely extend to economic considerations.

Simultaneously in R1, the imperative to satisfy regulatory requirements—which emphasize DTx safety and efficacy demonstrations for market approval—reinforces research organizations’ singular focus on clinical validation. This creates a self-reinforcing cycle that perpetuates the almost exclusive prioritization of clinical validation in institutional evaluation frameworks, despite growing literature recognition of economic evidence importance. While clinical validation represents a necessary milestone, it constitutes a short-term end point relative to the ultimate goal of successful DTx implementation in clinical practice.

Two additional observations merit attention. First, while EUT would predict comprehensive integration of regulatory requirements throughout development, the findings reveal these considerations often emerge much later. As Cheah (P13) noted:

A lot of times, the solution solves a problem, but it doesn’t end up somewhere because considerations of regulatory and reimbursement, with the actual greater landscape, are not in our minds.

The satisfaction of the regulatory requirements tends to be considered more as a chronological end point of clinical validation, rather than serving as guidance throughout the DTx development process.

Second, participants noted a “regulatory gap” for DTx, which may explain the limited consideration given to regulatory aspects by researchers early in the DTx process. For these reasons, the relationship between the satisfaction of the regulatory requirements and the level of economic consideration is depicted as a dashed line in Figure 2.

The positioning of research organizations on this translational spectrum is also largely influenced by the available funding sources, such as research grants, subsidies, donations, or investor capital. This influence is particularly significant regarding the comprehensiveness of their requirements (ie, how extensive the elements required in a grant proposal are, whether they include the clinical, technological, and economic impact of a potential DTx). As Emma (P1) noted:

Some grants now require us to think about commercialization pathways, which forces us to consider the economics earlier on.

Similarly, Camirah (P15) observed:

More and more, I find that these grant application forms require you to justify the economics of your innovation ... So to justify that, we always have to tell them how much economic benefit our solution would provide, which is hard to find.

While some funding bodies require cost impact assessments for DTx solutions, participants described this as an emerging rather than established practice.

These funding sources, through their requirements, can either enable or constrain action, influencing researchers’ consideration of various aspects in DTx development. In the causal model, the comprehensiveness of funding requirements represents a potential intervention point that could disrupt the R1 loop by introducing economic considerations into performance metrics, though this effect appears limited in current practice.

#### System Implications and Propositions

R1 and the impact of current requirements of funding mechanisms create persistent patterns that institutionalize clinical outcome prioritization while marginalizing complementary value dimensions necessary for sustainable implementation (Figure 2). Hence, it is posited:

- Current organizational settings, funding, and regulatory requirements collectively establish a rigid evaluative framework that systematically and exclusively prioritizes clinical evidence, effectively precluding the integration of alternative value dimensions, particularly economic considerations, throughout the DTx development process.
- The temporal delay between satisfying the regulatory requirements and the level of economic considerations creates a structural barrier that persists even when funding bodies begin to incorporate implementation-related factors into their awarding processes.
- Organizational settings, toward more translational aspects, that diversify team composition beyond clinical expertise will demonstrate greater integration of economic considerations throughout the DTx development lifecycle.
- Interventions targeting the weak causal links between both the regulatory and funding environments to the level of economic considerations offer potential leverage points for system change.

The professional norms mechanism functions primarily as a “situational mechanism” [82]. It describes how macrolevel contextual conditions and institutional power dynamics, which reflect embedded health care norms, influence DTx development approaches, predominantly driven by clinical considerations. The power of this mechanism lies in its ability to shape the decision space available to researchers before individual factors come into play, effectively defining the range of considerations perceived as legitimate or necessary. The subsequent mechanism reveals how intrapersonal factors and cognitive constraints further shape the way researchers make decisions in this predefined space.

#### The Researcher Experience Mechanism

The second mechanism (Figure 3) demonstrates how researchers’ professional background, including their training, experience, and expertise, alongside cognitive biases, create two reinforcing loops (R2: literacy loop and R3: role boundary loop) and one balancing loop (B1: bias loop) that systematically channel focus toward familiar domains and away from economic considerations.

The participant interviews revealed how researchers’ professional backgrounds systematically shape their approach to DTx development in ways that often exclude economic considerations, with researchers across different disciplines consistently acknowledging that their specialized training created blind spots regarding health economics perspectives. Ezra (P3), who has a science-heavy educational background, noted the absence of economic concepts in their formal training. These knowledge gaps are further compounded by cognitive biases shaped by professional training that influence decision-making throughout the DTx development process. Engineering-trained researchers like Eisa (P4) recognized their tendency to prioritize technical optimization over economic feasibility and broader health system integration requirements, while Sasha (P6) candidly summarized:

This is how it works [referring to the DTx development process], what you do, and your choices are partially driven by your past experiences.

In Figure 3, the researcher’s level of professional specialization serves as a fundamental determinant that shapes how narrowly focused their roles become (positive relationship in R3), influences their economic literacy level (negative relationship in R2), and contributes to the formation of cognitive biases (positive relationship in B1). This variable, bolded in Figure 3, indicates the centrality of this node, being in each of the three loops that constitute this mechanism.

#### Reinforcing Loops R2 and R3: Literacy and Role Boundary Loops

The study revealed indeed that researchers’ training and educational backgrounds—whether in engineering, clinical, or social sciences—created distinct mental models for approaching DTx development. Rather than optimizing utility or outcomes across all value dimensions, researchers demonstrated strong domain-specific thinking patterns. Eisa (P4) candidly acknowledged that it might create tunnel vision:

Because from my engineering background, I will not see things from a health economics point of view.

This domain-specific focus was reinforced by Ezra (P3):

Maybe it’s just me, like most of the classes I took, they were more like tech or science-heavy. I don’t think they ever mention anything regarding a translational type of work.

While Cheah (P13) noted some exposure to economic concepts during his specialization training, this proved to be an exception rather than the norm.

These statements illustrate how the R2 and R3 loops begin with the level of professional specialization and create path dependencies that shape decision frameworks. Professional training creates expertise in narrow fields, often accompanied by pressures to publish in specialized journals and secure domain-specific funding. This leads researchers to prioritize familiar domains (clinical or technical), thereby reinforcing the tendency to specialize further in those same domains. This creates a reinforcing loop where specialized expertise further narrows focus, decreasing the likelihood of acquiring broader economic competencies.

Within the R2 loop, the observed limited level of economic literacy among researchers, created or reinforced by the level of professional specialization, represents a key intermediate variable that creates persistent knowledge gaps in DTx development. It establishes a self-reinforcing cycle where researchers gravitate toward familiar domains and methodologies, whether clinical or technological. As noted earlier, while EUT suggests that researchers aim to complement their expertise by incorporating economic considerations, observations instead reveal satisficing behavior when faced with unfamiliar economic aspects. This behavior further strengthens the tendency to stay within familiar areas and exhibits a negative relationship with the level of cognitive biases: the higher the researcher’s level of economic literacy, the less likely they are to engage in satisficing behavior and the fewer cognitive biases they exhibit in this domain (Multimedia Appendix 1 [23-51]).

Within the R3 loop, the level of professional specialization strongly influences the narrowness of the researcher’s role as discussed above*,* which is further shaped by the degree of organizational translational research focus and the comprehensiveness of funding requirements.

As noted earlier, research institutions evaluate research-related performance metrics and incentives around publications and clinical validation. These metrics serve as visible manifestations of underlying professional norms and effectively constrain researchers’ roles and foci. They systematically exclude consideration of translational aspects or alternative value forms, such as economic utility, and they marginalize the implementation-oriented knowledge generation that could facilitate broader DTx adoption. Such metrics, while creating what EUT would consider a rational framework for maximizing utility within institutional constraints, potentially introduce what behavioral economists term “reward substitution” [83,84]. This phenomenon occurs when proximate goals substitute for ultimate goals. In this context, researchers focus on publications and clinical validation (proximate goals) rather than successful broad DTx implementation for improved clinical outcomes beyond limited trial participants (ultimate goals).

Consequently, two tensions emerge. First, academic publication and patenting can conflict due to their opposing requirements regarding public disclosure. Public disclosure can directly impact a researcher’s ability to patent an invention, as most patent systems require “novelty”—meaning the invention must not have been disclosed publicly before filing the patent application. Second, a potential and interesting tension exists where rational responses to institutional incentives may produce suboptimal outcomes from a broader utility perspective. As engineer-researcher Emma (P1) explained:

As a researcher, my key performance indicators are that I need to develop something, I need to publish something ... the time that I have to implement it is not in my key performance indicators.

This statement illustrates how the R3 loop operates in practice, with performance metrics directly shaping researcher priorities in ways that exclude economic considerations.

#### Balancing Loop B1: Bias Loop

The B1 loop in the causal model demonstrates how the level of cognitive biases, reinforced by researchers’ specialized expertise and a gap in economic literacy, systematically influences development decisions. Surya (P10) attributed these biases also to the consequences of professional training (déformation professionnelle in BDT terms), which offered limited to no exposure to economic principles, which further reinforces these biases. Sasha’s (P6) earlier statement about “This is how it works, what you do, and your choices are partially driven by your past experiences” captures how the B1 loop operates in practice, with past experiences shaping current perceptions and decisions. From the interviews, these biases manifested in various forms in DTx development decision-making, ranging from optimism bias regarding technical solutions, confirmation bias in clinical efficacy assessments, and status quo bias favoring existing health care delivery models:

Engineers often have unrealistic expectations about adoption rates because they underestimate institutional resistance to new technologies.

Surya, P10

In addition, Sasha (P6) mentioned:

I know from my professional experience ... that potential end users, such as patients, [in a feedback session] don’t tell the truth, might not care, or pay attention. The data will be seriously tainted if you just ask them straightforward questions.

This statement—based on professional experience—reveals the preconceived notion that participants are unreliable, a cognitive bias. Such beliefs may “seriously taint” (to use Sasha’s own terminology) the interpretation of future patient interactions, potentially leading to confirmation bias in data interpretation.

While these cognitive patterns are expected and appear throughout the DTx development process, they seem to systematically obscure economic considerations. They represent domain-specific expertise that shapes how researchers evaluate evidence and approach decision-making throughout the DTx lifecycle. They are identified in Figure 3 as negative influences on the level of economic considerations. The power of the balancing loop B1 lies in its largely unconscious operation, with researchers often unaware of how cognitive biases shape their perception of what constitutes relevant considerations. Emma (P1) illustrated this phenomenon:

Engineers think this is the problem and they work on a solution for it and then after they finish the solution when they’re trying to implement it, they realize that that’s actually not the problem; there’s something else.

#### System Implications and Propositions

The interaction between loops R2, R3, and B1 and their set of interdependent variables creates a particularly robust system resistant to change, one that maintains the observed gap in economic consideration throughout the DTx lifecycle. As Figure 3 demonstrates, when domain-specific training combines with cognitive biases, the effect is not merely additive but multiplicative, creating what SD identifies as a “success to the successful” archetype [73,74]. This dynamic generates momentum through reinforcing loops, establishing patterns where initial specialization becomes increasingly strengthened over time. This self-reinforcing mechanism can be moderated through the balancing loop B1, with carefully designed interventions that target these causal relationships.

These interdependent variables create a robust researcher experience mechanism influencing the timing and depth of economic considerations in DTx development. While the professional norms mechanism operates at the institutional level to constrain the decision space, this second mechanism operates at the individual level to shape how researchers navigate within that space. While researchers demonstrate rational decision-making within their domain expertise, they exhibit satisficing behavior when encountering unfamiliar economic considerations. The implications of this mechanism for DTx development are important; hence, it is posited:

- The more specialized a researcher’s training in clinical or technical domains, the greater the likelihood they will exhibit satisficing behavior when confronted with economic considerations in DTx development.
- Researchers’ cognitive biases, particularly domain-specific thinking patterns, will systematically influence their perception of what constitutes relevant evidence in DTx development, with economic evidence being reported as being systematically obscured.

The researcher experience mechanism represents an “action formation mechanism” [82]. It explains how researchers’ characteristics generate specific actions and decisions in DTx development. Unlike the professional norms mechanism, which shapes the broader decision context, this mechanism explains how individual researchers process information and make decisions within that context. The analysis demonstrates how these individual-level factors lead researchers to prioritize familiar domains, creating systematic patterns in DTx development approaches.

The investigation revealed a third critical mechanism related to the uncertainties of DTx adoption within health care systems. This adoption uncertainties mechanism addresses how researchers navigate the complex landscape of implementation challenges and stakeholder engagement throughout the DTx lifecycle, revealing a different dimension of barriers to economic consideration in DTx development. It operates in conjunction with the previous two, as illustrated by the cross-mechanism relationships in Figure 4, where elements from each mechanism ultimately influence economic considerations through various direct and indirect pathways.

#### The Adoption Uncertainties Mechanism

Implementation uncertainties surrounding DTx adoption within health care systems indeed emerge as a third critical mechanism shaping researchers’ development decisions. This mechanism (Figure 4) reveals how researchers’ awareness of adoption challenges drives increased focus on clinical needs, while stakeholder engagement simultaneously influences development approaches. Sara (P7) articulated this dynamic:

The big hurdle we see is that clinicians are the main influence on product acceptance. To have a product accepted by health professionals, you must address a real problem and consider workflow integration. If a physician has only seven minutes per patient but needs ten minutes to explain app installation, with weekly follow-ups and no additional payment, it won’t work. This must be considered from day one throughout development stages.

Sara’s (P7) insights suggest that DTx development processes should more systematically integrate real-world implementation constraints, recognizing that successful adoption ultimately depends on addressing genuine clinical problems within practical health care delivery contexts. Similar to the researcher experience mechanism, this mechanism emerges from the interplay between balancing and reinforcing forces, creating a complex system of development influences.

#### Balancing Loops B2a and B2b: Implementation-Driven Development and Stakeholder-Driven Development Loops

The awareness of implementation considerations serves as a central imperative throughout the DTx development process. It functions as the primary driver in both the B2a/b and R4 loops in the causal model. In the interviews, researchers consistently emphasized workflow integration, resource requirements, and organizational readiness. Practitioner-researchers confirmed implementation considerations as critical determinants of DTx success, reflecting both rational planning and practical responses to uncertainty in complex health care environments:

Focus [during implementation] should be on widespread adoption rather than profit maximization.

Cheah, P13

The findings reveal that greater researcher awareness of implementation adoption challenges positively associates with an increased perceived importance of unmet needs validation, ensuring future DTx interventions address specific unmet needs and reduce instances of having a “solution looking for a problem.”

Participants reported conducting consultation focus groups with patients and physicians, but revealed these sessions often narrowly target clinical unmet needs rather than broader implementation challenges:

We should also consider complex approval processes, challenges with pilot projects and sustainable funding, or yet the resistance to change from health care professionals...

Priscillah, P16

Although literature frames this step as “problem-specification” for identifying real-world needs [10], the participants’ narrower interpretation represents another manifestation of bounded rationality, where clinical validation takes precedence over comprehensive value assessment. This focus on clinical unmet needs, while important, often fails to incorporate broader economic dimensions necessary for successful implementation.

As the perceived importance of unmet needs validation grows, it reshapes research priorities and considerations beyond simple technological requirements, ultimately slowing the DTx technological development pace (negative feedback structure). Researchers respond by breaking the DTx development process into more manageable sequential DTx processes as a step-by-step approach. They spend more time ensuring their technology can address the unmet need—despite growing recognition of the need for more integrated approaches.

This sequential approach temporarily reduces uncertainty by emphasizing short-term, step-by-step linear progression rather than iterative development. However, this creates potential long-term integration challenges. While some researchers like Santana (P11) advocated for a “DTx implementation-aware development,” where adoption considerations actively shape early decisions, most researchers reported having a more linear and sequential approach, which contributes to the persistent gap between clinical validation and economic consideration. This is visualized as a green line in Figure 4, where the level of DTx process sequentiality tends to delay the level of economic considerations to later stages, beyond the “responsibilities” of the interviewed researchers, to use their own words. This sequential approach further delays implementation considerations (negative relationship). It creates a balancing effect in the system, counteracting the reinforcing dynamic of loop R4, and contributes to the system’s stability.

#### Reinforcing Loop 4 (R4): Collaboration Loop

In R4, the importance placed on addressing clinical needs creates a positive feedback dynamic, driving the level of stakeholder engagement, with a more multidisciplinary and interdisciplinary approach, acknowledged as crucial by the participants for successful DTx development. The interviews identified a complex network of stakeholders potentially engaged throughout the DTx lifecycle, including engineers, data scientists, behavioral scientists, economists, health care providers, clinicians, hospital administrators, regulatory bodies, and commercial stakeholders.

These collaborations enhance the awareness of implementation considerations, which ultimately reinforce the perceived importance of unmet needs validation (positive relationship, completing the R4 loop). However, rather than following EUT’s prediction of comprehensive stakeholder integration, the interviews show that stakeholder engagement patterns typically follow a more sequential approach. This results in what participants characterize as delayed stakeholder engagement, which is reinforced by the negative influence of the level of DTx process sequentiality. This challenge is illustrated by Camirah’s (P15) reflection:

I initially did not involve hospital administrators in my solution development, which created later challenges in the implementation process.

When the R4 loop functions suboptimally due to limited stakeholder engagement—as in Camirah’s (P15) example—it may fail to address the full complexity of implementation, including economic considerations.

#### System Implications and Propositions

The dynamic tension between loops B2a/b and R4 in Figure 4 creates what is called in SD a “fixes that fail” archetype [73,74]. Short-term solutions, such as sequential development to address a clinical unmet need, create longer-term consequences like fragmentation and delayed stakeholder engagement. While ensuring a DTx addresses clinical needs is important, this approach produces an ongoing tension between immediate development priorities and long-term implementation success. Researchers increasingly recognize the need for early stakeholder collaboration to address adoption uncertainties. However, translating this recognition into systematic practice remains a key challenge in DTx development. Hence, it is posited:

- The greater the perceived implementation uncertainty surrounding a DTx, the higher the likelihood that researchers will seek a more multidisciplinary approach, integrating various stakeholder feedback throughout the DTx development process.
- DTx development projects with formalized processes for early stakeholder feedback across diverse stakeholder groups will demonstrate higher levels of economic consideration throughout the development process.
- The temporal disconnect between the level of DTx process sequentiality and the level of economic considerations creates structural barriers that persist even when researchers recognize the importance of implementation factors.

This mechanism represents a “transformational mechanism” [82]. It shows how researchers’ responses to implementation uncertainties and their decisions about stakeholder engagement collectively transform into broader patterns in DTx development practices, ultimately aggregating into systematic patterns of sequential development processes and delayed stakeholder engagement.

While the first two mechanisms primarily explain why economic considerations are marginalized in DTx development, the adoption uncertainties mechanism helps explain why implementation concerns (which often have economic dimensions), even when recognized, tend to be addressed in ways that are clinical as one priority, while further fragmenting the development process rather than promoting integrated approaches.

#### Cross-Mechanism Relationships and Secondary Loops

**Figure 5:**
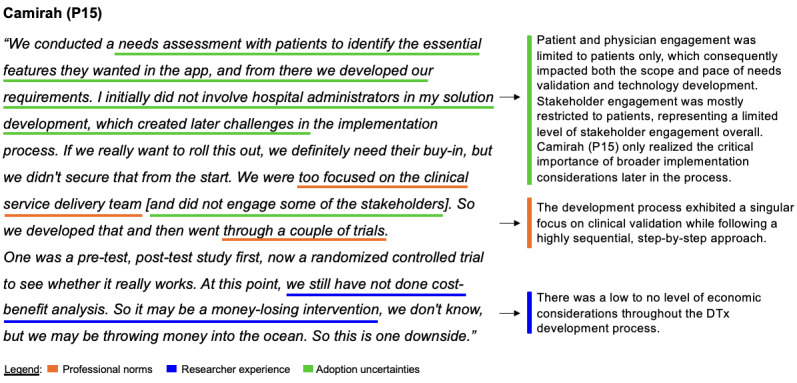
Cross-mechanism interactions illustrated through Camirah (P15)’s quote. This figure illustrates cross-mechanism interactions. Using participant Camirah (P15)’s shared experience, this example demonstrates how the three identified mechanisms are simultaneously at play, manifest, and interact, showing how clinical validation focus, limited stakeholder engagement, and economic considerations intersect in the development process.

Beyond the primary feedback loops that characterize each of the mechanisms, the analysis reveals six secondary loops (B3a/b/c, B4, and B5a/b). These are detailed in Multimedia Appendix 11 and integrated into the CLD in Multimedia Appendix 12. The loops function as crucial integrative elements between these mechanisms. While exerting less direct influence than primary loops, these cross-mechanism relationships operate through “contingent causality,” where outcomes vary based on interactions with prior effects from other mechanisms [31]. They demonstrate how effects propagate throughout the system, creating an interconnected web of causality that ultimately shapes economic considerations in DTx development (see Figure 5 for an illustration of these cross-mechanism interactions). By identifying these secondary loops, the analysis transcends mere pattern recognition to reveal the underlying generative mechanisms at work, highlighting both the complexity of researchers’ decision frameworks and potential intervention points where development practices might be modified to better integrate economic considerations.

## Discussion

### Main Findings

This study advances understanding of researchers’ economic decision-making in DTx development through the lens of CR, revealing complex interactions between institutional structures, researchers’ intrapersonal factors, and implementation uncertainties. By identifying three interrelated generative mechanisms—professional norms, researcher experience, and adoption uncertainties—the research provides a comprehensive explanatory framework for the systematic deprioritization of economic considerations observed in DTx development, despite their acknowledged importance for successful implementation. By integrating insights from both EUT and BDT, the findings demonstrate how researchers navigate complex decision environments. They seek to optimize outcomes within their domain of expertise, while exhibiting satisficing behavior when they encounter economic considerations outside their professional training. Rather than reflecting a simple absence of economic thinking, our analysis reveals a more nuanced reality: formal economic considerations remain peripheral, while substantive economic decision-making occurs implicitly throughout DTx development.

This apparent paradox emerges because economic considerations in DTx development operate on two distinct levels: (1) explicit economic considerations—direct cost-effectiveness analyses, budget impact assessments, and formal economic evaluations that researchers consciously recognize as “economic”—and (2) implicit economic considerations—implementation-related factors that have economic implications but are not conceptualized by researchers as economic decisions.

These three mechanisms collectively reveal what could be termed as an “economic awareness gap”: a systematic disconnect between researchers’ actions and their conceptual frameworks. This gap manifests in three important ways: (1) terminological disconnection—researchers use economic reasoning without economic vocabulary, (2) disciplinary boundaries—implementation concerns are viewed as separate from economic considerations, and (3) temporal fragmentation—economic implications are recognized but not integrated across development phases. Understanding this gap provides crucial insights for developing more effective strategies to bridge the divide between technical innovation and economic sustainability in DTx development.

### Comparison With Prior Work

The findings both validate and expand upon existing literature on health care innovation decision-making, especially about decision frameworks’ tendency in early development phases to prioritize technical feasibility and clinical applicability, with economic considerations often introduced later in the development cycle [85]. Several studies analyzed decision-making in health care and emphasized a tendency toward focusing on short-term results and using a single-performance measure for problems in the decision-making process [68]. This observation echoes current research findings regarding researchers who prioritize short-term end points, such as clinical validation. The literature highlights how well-intentioned interventions and inherent trade-offs might generate ripple effects throughout the system, extending far beyond the isolated and more short-term consequences that stakeholders might initially anticipate [67].

The predominance of clinical considerations in this study aligns with previous research on DTx technology development [7,22], while the identification of specific mechanisms through which economic considerations become systematically deprioritized advances the understanding of researcher decision-making processes. The findings on the influence of professional training on decision frameworks corroborate earlier research about educational background in innovation development [86-88]. Additionally, the analysis of adoption uncertainties provides novel insights into how implementation concerns shape early DTx development decisions. The significance found regarding adoption considerations is consistent with existing literature documenting implementation challenges in digital health interventions [5,6,60].

These findings suggest several actionable approaches for different stakeholders, offering intervention points to enhance economic considerations throughout DTx development. As more evidence emerges about the potential cost-effectiveness of DTx across diverse therapeutic areas [17,89-91], these findings become increasingly relevant for strengthening the economic impact of these interventions. This creates a foundation for more integrated approaches to value assessment in digital health innovation.

Translational research organizations should adopt structured development frameworks incorporating economic assessments at multiple stages, systematically diversify team composition to include economic expertise from project inception, and revise evaluation metrics to include implementation feasibility indicators. Individual researchers would benefit from structured decision-making frameworks that explicitly consider economic factors, simplified economic assessment tools, targeted training in health economic principles, and early implementation planning with stakeholder engagement. For policy makers and funding agencies, developing standardized economic evaluation guidelines specific to DTx creates incentives for early economic consideration through funding mechanisms. This approach would support implementation research and promote more integrated approaches to value assessment in digital health innovation.

### Limitations

When interpreting these research findings, it is important to acknowledge certain limitations. Methodologically, while the sample included experienced researchers from diverse backgrounds, participants were predominantly, at the time of the study, evolving in academic and public health care contexts. This sampling approach enabled detailed examination of mechanisms within publicly funded research environments, which constitute a significant proportion of global DTx development activities and serve as foundational sites for innovation translation [92]. Moreover, commercially oriented DTx development environments, characterized by profit-driven objectives and investor accountability requirements, may operate under different institutional logics that could alter the identified mechanisms. However, these findings maintain practical relevance for commercial settings through the prevalent academic-commercial collaborations that characterize DTx development [92], particularly during clinical validation phases where academic expertise remains paramount. Future research should validate the mechanisms’ generalizability across these diverse institutional contexts.

From a geographic perspective, the sample, while international, had greater representation from specific regions, which may not fully capture contextual factors unique to specific health care systems. Additionally, the recruitment approach may have introduced selection bias, attracting participants with a greater interest in economic considerations, although the findings of limited economic integration suggest this is not a genuine concern.

From a theoretical perspective, CR emphasizes that generative mechanisms often are “context-sensitive” and operate contingently [93,94]. This implies that the mechanisms identified in this study could, but not necessarily, produce analogous structures, but inevitably exhibit different behavioral outcomes in varied organizational, regulatory, or funding contexts, as discussed above.

The CLDs, while systematically developed through a triangulation process both of data and of methods, do represent the available evidence, but perhaps not fully the nuanced and textured complexity of real-world decision processes. The exploratory qualitative approach used is valuable for identifying key relationships, but it does limit the ability to assign quantitatively the weighted importance of different causal relationships or the strength of feedback mechanisms.

Even with these limitations, the rigorous methodological approach used, theoretical grounding, and consistent findings across a diverse set of experienced participants suggest that the identified mechanisms represent robust explanatory frameworks structured for understanding economic considerations in DTx development.

### Future Research

This study opens several promising avenues for future investigation. Methodologically, the identified mechanisms suggest feasible intervention points that could be tested through both pragmatic and hypothetico-deductive designs. Mixed methods case studies following specific DTx projects through their development lifecycle could provide detailed process evaluations of how economic training interventions affect researcher decision-making, using interviews, ethnographic observations of team meetings, and analysis of project documentation to track changes in decision tipping points.

Longitudinal research approaches could provide particularly critical insights into mechanism evolution over time through two key study designs. Researcher trajectory studies following individual researchers across multiple DTx projects over 2-3 years could reveal how decision-making patterns evolve with experience and training, particularly examining whether exposure to economic considerations in one project influences behavior in subsequent projects. Institutional evolution studies could track how organizational changes—such as new policies, key performance indicators, modified team structures, or updated funding requirements—affect the three identified mechanisms over time, providing evidence of whether interventions create sustained behavioral change or merely temporary compliance with new requirements.

Complementing these longitudinal approaches, survey-based validation could use proven instruments measuring economic literacy and decision-making approaches among researchers before and after targeted educational interventions, supplemented by qualitative interviews exploring changes in decision-making processes.

### Conclusions

This study reveals three interrelated generative mechanisms that systematically deprioritize economic considerations despite their recognized importance. The professional norms, researcher experience, and adoption uncertainties mechanisms operate through complex feedback loops that create persistent patterns resistant to change, explaining why economic considerations remain peripheral even as their importance becomes increasingly acknowledged in the literature.

The identification of these mechanisms provides valuable intervention points for better integrating economic considerations throughout the DTx lifecycle and enhancing development processes toward more comprehensive value assessment. Ultimately, addressing these underlying mechanisms could improve the likelihood of DTx solutions achieving both clinical efficacy and economic sustainability, thereby enhancing their potential for widespread adoption and meaningful impact on health care systems.

## Appendix

Multimedia Appendix 1 Integrated theoretical framework.

Multimedia Appendix 2 Demographic data of study participants.

Multimedia Appendix 3 COREQ checklist.

Multimedia Appendix 4 Interview topic guide.

Multimedia Appendix 5 Data analysis methodology summary.

Multimedia Appendix 6 Final codebook.

Multimedia Appendix 7 Saldaña's cycles of coding methodology.

Multimedia Appendix 8 Illustrative quotes for the three generative mechanisms.

Multimedia Appendix 9 Triangulation approach to validating mechanisms and mapping cause-effect relationships in DTx decision-making.

Multimedia Appendix 10 Detailed results from steps 1 and 2 of data analysis.

Multimedia Appendix 11 Overview of individual primary and secondary balancing and reinforcing loops in the CLD.

Multimedia Appendix 12 Primary within-mechanism and secondary cross-mechanism loops in DTx development.

## Abbreviations

BDT: behavioral decision theory
CLD: causal loop diagram
COREQ: Consolidated Criteria for Reporting Qualitative Research
CR: critical realism
DT: decision theory
DTx: digital therapeutics
EUT: expected utility theory
QSD: qualitative systems dynamics
SD: systems dynamics

## Data Availability

All data generated or analyzed during this study are available from the corresponding author on reasonable request.
