# Supplementary material for: Understanding Economic Decision-Making in Digital Therapeutics Development: Qualitative Approach
Source: J Med Internet Res. 2025 Sep 16;27:e79746. doi: 10.2196/79746 (PMC12485261; doi:10.2196/79746)

This appendix presents a comprehensive synthesis of the literature review that underpins the proposed theoretical framework guiding this study. The integration of Decision Theory (DT) and Systems Thinking (ST) within a Critical Realism (CR) paradigm provides robust analytical tools for examining complex decision-making processes in DTx development while informing the methodological approach employed in this research.

**Critical Realism**The study is grounded in CR, which provides a philosophical framework recognizing three interconnected domains of reality [23]:

- **Empirical**: This domain captures the perceptions, feelings and experiences of the researchers as they navigate the DTx development process. It represents only the most surface level of the observable reality.
- **Actual**: This domain refers to events or phenomena that occur, independently of the researchers' awareness or interpretation. While these events are concrete occurrences, they emerge from deeper causal powers.
- **Real**: This domain contains the causal mechanisms that generate observable events in the “actual” and shape experiences. These mechanisms operate whether they are activated or perceived, such as institutional pressures for commercialization manifested through performance metrics, underlying power dynamics between academic and commercial interests, or structural economic constraints that influence priorities. These mechanisms, while not directly observable, can be theoretically identified through their manifestation in events and experiences.

This approach enables exploration beyond surface-level patterns to identify deeper explanatory mechanisms shaping researchers' decision-making [70]. Recent research has also demonstrated CR particular relevance for social studies of health, illness, and medical settings [71,72], as well as in information systems research [42,46]. Having established CR as the research study’s philosophical foundation, the theoretical frameworks can now be examined more in-depth to understand how researchers navigate the complexities of DTx development.

**Decision Theory in Healthcare Innovation**DT provides a comprehensive foundation for analyzing complex choice-making processes under uncertainty – a central challenge in healthcare innovation. DT helps us understand both the observable patterns in researchers' choices and the underlying mechanisms generating them by drawing on principles from economics [44], psychology [73], statistics [74], and medicine [75]. In healthcare innovation, DT encompasses both normative and descriptive approaches [25].

***Expected Utility Theory (EUT) - The Normative Component***EUT, developed by von Neumann and Morgenstern [76], offers valuable insights into optimal decision-making under uncertainty—a persistent challenge in healthcare [77,78]. EUT posits that individuals make decisions by evaluating the probability and utility (satisfaction or value) of each possible outcome. The theory expresses rational choice mathematically as the maximization of expected utility, calculated by multiplying the utility of each outcome by its probability, providing a weighted average of utility outcomes while accounting for inherent risks and uncertainties of DTx development. In healthcare, this manifests through structured evaluation frameworks like Health Technology Assessment (HTA), which provides a systematic approach to evaluating new technologies by weighing potential benefits against costs.

Despite its value, EUT faces several limitations in real-world healthcare settings [79]. The assumption of rationality conflicts with bounded rationality, as healthcare professionals often work with incomplete or ambiguous information. Precise utility quantification is challenging in healthcare's multidimensional outcomes. Multiple stakeholders with diverse interests challenge unified utility maximization. The theory also struggles to account for heuristic decision-making in healthcare practice [80]. These limitations suggest that while EUT provides valuable theoretical insights and a structured framework for decision analysis its practical application must be complemented by approaches that account for healthcare decision-making complexities.

***Behavioral Decision Theory (BDT) - The Descriptive Component***BDT examines how researchers actually make decisions, acknowledging inherent human biases and limitations. Key concepts include bounded rationality [81,82], where decision-makers select satisfactory rather than optimal solutions under cognitive constraints; cognitive biases such as optimism bias [83], pro-innovation bias [84], and selection bias [85]; and heuristics as mental shortcuts used to navigate decisions under uncertainty [24,86].

BDT also has limitations in healthcare contexts. It may not fully capture institutional and systemic factors [87]. Regulatory frameworks often override individual behavioral tendencies. It may also underestimate the value of professional experience and domain expertise in healthcare settings [88].

As Desmond et al note, decision-making in healthcare innovation encompasses various strategies employed under different circumstances [24]. The integration of normative and descriptive components creates a robust theoretical foundation that captures such complexity by defining optimal decision-making approaches while accounting for human tendencies and real-world complexities in healthcare innovation (Table S1). This dual perspective is particularly relevant when examining how researchers navigate economic considerations in DTx development, where decisions emerge from an intricate balance between rational analytical approaches (EUT) and the inevitable influence of behavioral and contextual factors (BDT).

**Table S1.** Relevance of decision theory in the context of the research question.

| **Theoretical Foundation** | **Core principles** | **Relevance to the Research Question** |
| --- | --- | --- |
| Expected Utility Theory (EUT) | - Rational decision-making based on utility maximization; - Systematic quantification of utility (e.g., health outcomes) through HTA; - Evaluation of risk and uncertainty. | - Provides a framework for rational decision-making under uncertainty, offering standardized approaches for generating and evaluating evidence; - Guides the evaluation of technological and clinical factors using economic models (such as HTA) to maximize utility and optimize health outcomes; - Informs decision-makers in resource allocation during technology development and validation. |
| Behavioral Decision Theory (BDT) | - Cognitive biases and decision heuristics; - Bounded rationality; - Contextual dependence of decisions. | - Cognitive biases may lead researchers to overestimate benefits or underestimate risks, skewing development decisions; - Affects how economic risks and clinical uncertainties are perceived, potentially influencing technology development and prioritization; - Highlights the importance of understanding the decision context and available information to distinguish between rational and satisfactory decision-making. |

**Systems Thinking: Extending Decision Theory**While DT provides valuable insights into individual decision-making processes, it does not fully capture the complex, multi-stakeholder nature of healthcare innovation. ST offers a complementary framework that situates individual decision-making within broader systemic contexts [26].

ST provides, indeed, a robust framework for understanding complex, multi-level systems where technological, individual, organizational, and broader contextual factors interact dynamically, influencing researchers’ decision-making processes and ultimately DTx development trajectories [89]. These interactions over time create emergent properties that cannot be understood by examining components in isolation, making ST particularly valuable for comprehending the complexity of researchers’ decision-making processes in DTx development.

ST introduces several essential concepts: emergence, where system behaviors arise from interactions between components; non-linearity, where minor decisions can produce disproportionately large effects; feedback loops, where actions produce consequences that influence subsequent decisions; power dynamics, where asymmetries between stakeholders influence which considerations receive priority; and temporal dynamics, where development unfolds across multiple time horizons [90].

ST, particularly through qualitative system dynamics (QSD) modeling, that originates from system dynamics (SD), allows us to identify and analyze the varying temporal scales of feedback loop structures in DTx development. By incorporating temporal dynamics, even if qualitatively, into the analysis, it reveals how the tension between short-term economic pressures and long-term value creation, as navigated by researchers, can be better understood.

QSD, a methodology within ST, offers structured approaches like Causal Loop Diagrams (CLDs) to map relationships and feedback loops between system elements. These methods help capture non-linear mechanisms and identify potential intervention points [26,27].

**Theoretical Framework Synthesis**The complex, multi-stakeholder nature of DTx development requires robust theoretical grounding to understand researchers' decision-making processes. DT, through its dual components of EUT and BDT, provides a comprehensive framework for analyzing both the underlying mechanisms and contextual conditions that influence researchers' decision-making. ST offers a natural extension to DT by providing conceptual tools to understand how individual decisions both influence and are influenced by broader system structures.

As illustrated in Figure S1, this study's integrated theoretical framework combines DT with ST within a CR ontology to comprehend researchers' decision-making processes. This integrated approach enables us to identify not just what economic decisions are made, but how they emerge from complex interactions between individual cognitive processes, organizational structures, and environmental contexts. Such understanding is crucial for identifying potential intervention points and developing more effective strategies for economically sustainable DTx innovations that successfully navigate the complex pathway from concept to clinical implementation.

**Figure S1.** Integrated theoretical framework for researchers’ decision-making in DTx development.


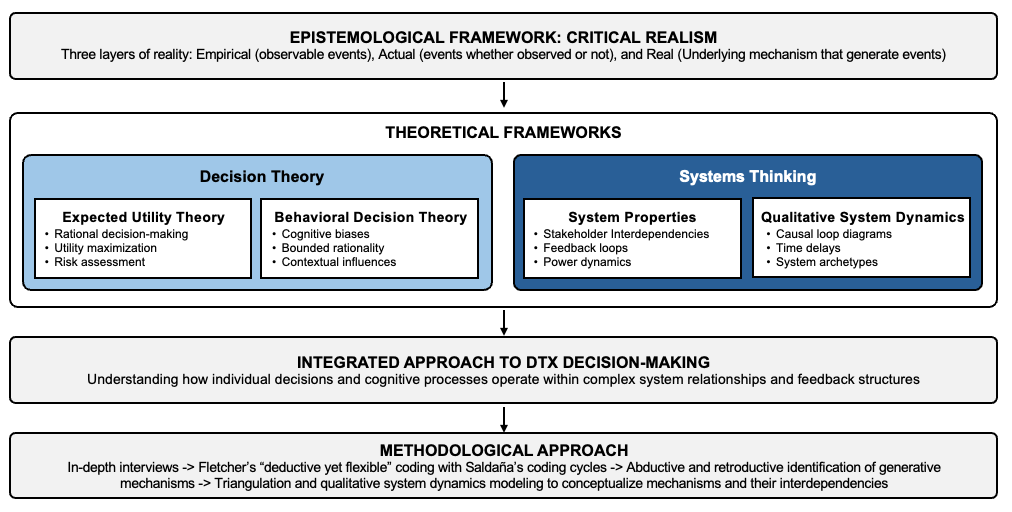

Supplement: Multimedia Appendix 1 [file jmir_v27i1e79746_app1.docx]
