# Supplementary material for: Understanding Economic Decision-Making in Digital Therapeutics Development: Qualitative Approach
Source: J Med Internet Res. 2025 Sep 16;27:e79746. doi: 10.2196/79746 (PMC12485261; doi:10.2196/79746)
Supplement: Multimedia Appendix 2 [file jmir_v27i1e79746_app2.docx]

| **Pseudonym^a^**  *(participant number)* | **Field of practice** | **Experience across the DTx lifecycle^b^** | | | **Years of experience** | **Setting** *(country)* | **Gender** | **Education** |
| --- | --- | --- | --- | --- | --- | --- | --- | --- |
|  |  | 1 | 2 | 3 |  |  |  |  |
| **RESEARCH-ENGINEERS** | | | | | | | | |
| Emma  *(P1)* | Biomedical engineering | x | x | x | > 10 | Public academic institution *(Singapore)* | F | Ph.D. |
| Eva  *(P2)* | Biomedical engineering | x | x | x | > 10 | Public academic institution  *(USA)* | F | Ph.D. |
| Ezra  *(P3)* | Biomedical engineering | x | x |  | > 5 | Public academic institution  *(Singapore)* | M | Ph.D. |
| Eisa  *(P4)* | Biomedical engineering | x | x |  | > 5 | Public academic institution  *(Singapore)* | M | Ph.D. |
| Elisabeth  *(P5)* | Neuroengineering | x | x | x | > 10 | Private healthcare organization  *(Switzerland)* | F | Ph.D. |
| **HEALTH SYSTEMS AND SOCIAL SCIENCE RESEARCHERS** | | | | | | | | |
| Sasha  *(P6)* | Behavioral sciences |  |  | x | > 10 | Public academic institution  *(Singapore)* | M | Ph.D. |
| Sara  *(P7)* | Healthcare management |  |  | x | > 10 | Public academic institution  *(Germany)* | F | Ph.D. |
| Senna  *(P8)* | Health policy |  | x | x | > 5 | Public academic institution  *(United Kingdom)* | M | Ph.D. |
| Shiva  *(P9)* | Health policy | x | x |  | > 5 | Public academic institution  *(Singapore)* | M | Ph.D. |
| Surya  *(P10)* | Health economics | x | x | x | > 20 | Public academic institution  *(United Kingdom)* | M | Ph.D. |
| Santana  *(P11)* | Health economics |  | x | x | > 10 | Public academic institution  *(Germany)* | M | Ph.D. |
| **CLINICIAN-RESEARCHERS** | | | | | | | | |
| Christoph  *(P12)* | Neuropsychology |  | x | x | > 20 | Private healthcare provider  *(Australia)* | M | Ph.D. |
| Cheah  *(P13)* | Ophthalmology | x | x |  | > 10 | Public healthcare provider  *(Singapore)* | M | Ph.D. |
| Chevannah  *(P14)* | Cardiology | x |  | x | > 10 | Private healthcare organization  *(Germany)* | F | Ph.D. |
| Camirah  *(P15)* | Nursing | x | x |  | > 5 | Public healthcare provider  *(Singapore)* | F | Ph.D. |
| **PRACTITIONER-RESEARCHERS** | | | | | | | | |
| Priscillah  *(P16)* | Technology implementation | x | x | x | > 20 | Public healthcare provider  *(Canada)* | F | M.Sc. |
| Priyah  *(P17)* | Technology implementation | x | x | x | > 15 | Public healthcare provider  *(Singapore)* | F | Ph.D. |

^a^The pseudonyms are designed to convey key information about each participant. The first letter indicates their role: ‘E’ for research-engineers, ‘S’ for health systems and social science researchers, ‘C’ for clinician-researchers, and ‘P’ for practitioner-researchers. The last letter denotes their current work setting: ‘A’ for academic organization, and ‘H’ for healthcare providers or organizations (either private or public). The gender of the pseudonym corresponds to the participant's sex. For instance, the pseudonym “Christoph” (P12) signifies a male clinician-researcher working in a healthcare organization or provider.

^b^Phase 1 encompasses the technological development of the DTx intervention, followed by phase 2, the clinical validation of the DTx intervention through clinical trials, and finally phase 3 corresponds to the implementation of the DTx intervention in a real-world environment.
