# Supplementary material for: Understanding Economic Decision-Making in Digital Therapeutics Development: Qualitative Approach
Source: J Med Internet Res. 2025 Sep 16;27:e79746. doi: 10.2196/79746 (PMC12485261; doi:10.2196/79746)
Supplement: Multimedia Appendix 3 [file jmir_v27i1e79746_app3.pdf]

COREQ checklist.

Item No	Guide Questions/Description	Comment
**Domain 1: Research team and reflexivity**		
**Personal Characteristics**		
1. Interviewer/ facilitator	Which author/s conducted the interviews?	YS conducted the semi-structured interviews.
2. Credentials	What were the researcher's credentials?	YS holds an M.Sc. in Entrepreneurship and Strategic Management and is a doctoral student researching economic value drivers for DTx. LMC has a Ph.D. in Strategic Management and Applied Economics and conducts research in Information Technology management. AM holds a Ph.D. in Management Science, specializing in decision analysis and operations research, with expertise in healthcare decision-making and health economics. DH holds a Ph.D. in Biomedical Engineering, SL in Communication and Information, and GS a Ph.D. in Economics.
3. Occupation	What was their occupation at the time of the study?	YS served as a Director at the Institute of Digital Medicine (WisDM), National University of Singapore, while LMC was a Professor in the Department of Analytics, Operations, and Information Technologies, School of Management Sciences, at the University of Quebec at Montreal, Canada. DH was the Provost's Chair Professor, Director of the Institute for Digital Medicine (WisDM), Director of the N.1 Institute for Health, and Head of the Department of Biomedical Engineering at the National University of Singapore. AM holds joint appointments at the National University of Singapore, Duke-NUS and the University of Strathclyde. SL and GS are both research fellows at the Institute of Digital Medicine (WisDM), National University of Singapore.
4. Gender	Was the researcher male or female?	One woman-identifying researcher and five male-identifying researchers.
5. Experience and training	What experience or training did the researchers have?	YS, LMC, AM, SL, GS, and DH have extensive experience in conducting qualitative health research. Additionally, YS, LMC, and DH have recently published relevant papers to the research question. LMC teaches research methods in the areas of information technology. AM focuses on developing methodologies for supporting complex healthcare resource allocation and policy decisions, having made significant contributions to health technology assessment and healthcare prioritization. SL and GS specialize in decision-making and behavior changes in health and public policy.
**Relationship with participants**		
6. Relationship established	Was a relationship established prior to study commencement?	YS and DH had varying relationships with the health community at large, while LMC, AM, SL, and GS had no relationships with participants prior to the study. All were perceived as unbiased researchers rather than persons with a particular stake, stance or opinion about the topic.
7. Participant knowledge of the interviewer	What did the participants know about the researcher?	The interviewers introduced themselves to participants and describing the context, scope and purpose of the research, going through the different sections of the consent form. The contents followed the exact guidelines as certified by ethics review boards for research with human beings of all institutions involved.
8. Interviewer characteristics	What characteristics were reported about the interviewer/facilitator?	The participants were informed of the knowledge gap and corresponding research question as context for the interview, highlighting the interviewers' interest in addressing this gap without presenting specific assumptions or potential biases. The contents followed the exact guidelines as certified by ethics review boards for research with human beings of all institutions involved.
**Domain 2: study design**		
**Theoretical framework**		
9. Methodological orientation and Theory	What methodological orientation was stated to underpin the study?	Critical realism was established as the study's philosophical foundation. Theoretical frameworks such as DT and ST were introduced to examine and interpret how researchers navigate the complex choices involved in DTx development.

Item No	Guide Questions/Description	Comment
**Participant selection**		
10. Sampling	How were participants selected?	Purposive sampling was used. Initial recruitment occurred through the research team's professional network, with participants encouraged to refer qualified colleagues through snowball sampling.
11. Method of approach	How were participants approached?	Email invitations were sent to individuals who could potentially meet the following inclusion criteria: (1) a minimum of five years of experience in DTx development or clinical validation, (2) current involvement in the technological development or clinical validation of a DTx, (3) fluency in English or French. The recruitment process followed the exact guidelines as certified by ethics review boards for research with human beings of all institutions involved.
12. Sample size	How many participants were in the study?	17 Participants. For details of the study participants' demographic data, please refer to Multimedia Appendix 2.
13. Non-participation Setting	How many people refused to participate or dropped out? Reasons?	While several individuals did not respond to the invitation, nobody explicitly declined the invitation. All those who agreed to participate remained committed throughout the study. This followed the exact guidelines as certified by ethics review boards for research with human beings of all institutions involved regarding participants' informed consent and right to withdraw without justification.
14. Setting of data collection	Where was the data collected?	Data were collected through either Zoom or face-to-face interviews and analyzed using ATLAS.ti software (ATLAS.ti Scientific Software Development GmbH).
15. Presence of nonparticipants	Was anyone else present besides the participants and researchers?	None.
16. Description of sample	What are the important characteristics of the sample?	Fully presented in Multimedia Appendix 2.
**Data collection**		
17. Interview guide	Were questions, prompts, and guides provided by the authors? Was it pilot tested?	A semi-structured interview guide with key questions was designed to explore topics including: (1) participants' roles as DTx researchers, (2) their priorities and key considerations throughout the DTx lifecycle, and (3) day-to-day prioritization and decision-making processes. Please refer to Multimedia Appendix 4 for details of the topics discussed.
18. Repeat interviews	Were repeat interviews carried out? If yes, how many?	No.
19. Audio/visual recording	Did the research use audio or visual recording to collect the data?	All interviews were audio-recorded and later transcribed. This followed the exact guidelines as certified by ethics review boards for research with human beings of all institutions involved in data management procedures during the research, during the analysis, and post-reporting.
20. Field notes	Were field notes made during and/or after the interview or focus group?	Following each interview, the interviewer documented brief notes highlighting the interview's nature and key themes emphasized by the interviewee.
21. Duration	What was the duration of the interviews or focus group?	Interview duration varied but typically ranged from 45 to 60 minutes.
22. Data saturation	Was data saturation discussed?	Yes.
23. Transcripts returned	Were transcripts returned to participants for comment and/or correction?	No.
**Domain 3: analysis and findings**		
**Data analysis**		

		Comment
24. Number of data coders	How many data coders coded the data?	The interviewer coded and described the data, regularly discussing the resulting codes with the research study team. The analysis combined deductive and inductive coding, followed by abductive and retroductive processes, to identify generative mechanisms underlying observed decision-making patterns.
25. Description of the coding tree	Did the authors provide a description of the coding tree?	Yes. Please refer to Multimedia Appendix 7 for the verbatim to codes, subthemes, and themes developed through Fletcher's "deductive yet flexible" coding [33] with Saldaña's coding cycles [38].
26. Derivation of themes	Were themes identified in advance or derived from the data?	Derived from the data.
27. Software	What software, if applicable, was used to manage the data?	ATLAS.ti software (ATLAS.ti Scientific Software Development GmbH).
28. Participant checking	Did participants provide feedback on the findings?	Discussions were scheduled with three DTx experts at different time intervals who met the study inclusion criteria but were outside the original sample to interpret, validate, and refine emerging versions of the CLD.
**Reporting**		
29. Quotations presented	Were participant quotations presented to illustrate the themes/findings? Was each quotation identified?	Yes.
30. Data and consistent findings	Was there consistency between the data presented and the findings?	SD modeling was applied to visualize causal relationships through triangulated data sources, ultimately aiming to achieve a higher degree of internal and external validity, strengthening confidence in the findings.
31. Clarity of major themes	Were major themes clearly presented in the findings?	Yes, major themes are clearly identified in the paper, as well as the generative mechanisms underlying observed researchers' decision-making patterns.
32. Clarity of minor themes	Is there a description of diverse cases or a discussion of minor themes?	No.
