# Supplementary material for: Understanding Economic Decision-Making in Digital Therapeutics Development: Qualitative Approach
Source: J Med Internet Res. 2025 Sep 16;27:e79746. doi: 10.2196/79746 (PMC12485261; doi:10.2196/79746)
Supplement: Multimedia Appendix 4 [file jmir_v27i1e79746_app4.docx]

| **Topics** | **Guiding topics for discussion** |
| --- | --- |
| Background and Opening Topics | - Professional background and role in DTx development - Current DTx project(s) context and stage - Professional training influences on economic decision-making approaches |
| Understanding of Economic Value  *(Real to Actual Domain)* | - Personal understanding of DTx economic value - Experience with economic decision-making in DTx projects - Key considerations, priorities and decision-making drivers that guide DTx development - Organizational context and constraints |
| Contextual Conditions  *(Actual Domain)* | - Market and regulatory environment - Resource availability and limitations - Stakeholder collaboration, influences and pressures - Organizational incentives and their influence - How uncertainty affects decision-making processes |
| Practical Implementation *(Empirical Domain)* | - Specific examples of economic decisions made - Implementation challenges and solutions - Success metrics and evaluation methods - Lessons learned from past decisions - Trade-offs between competing priorities - Balancing clinical versus economic considerations - Role of experience in decision-making - Group decision-making dynamics |
| Reflection on Practice | - Perceived effectiveness of current decision-making approaches - Challenges in translating economic considerations into practice - Tools or support needed for better decision-making - Recognition of potential biases or limitations - Evolution of the decision-making approach over time |
| Closing | - Additional thoughts on economic value considerations - Recommendations for other researchers - Reflections on ideal versus actual decision-making processes |
