# Supplementary material for: Understanding Economic Decision-Making in Digital Therapeutics Development: Qualitative Approach
Source: J Med Internet Res. 2025 Sep 16;27:e79746. doi: 10.2196/79746 (PMC12485261; doi:10.2196/79746)
Supplement: Multimedia Appendix 5 [file jmir_v27i1e79746_app5.docx]

| **Step** | **Description** | **Results** |
| --- | --- | --- |
| **1. Coding and identification of demi-regularities** | - Creation of a “deductive yet flexible” coding framework combining DT concepts with DTx literature, while effectively capturing CR's stratified ontology [33]. - Application of both deductive and inductive coding using ATLAS.ti software. - Implementation of Saldaña’s cycles of coding methodology for systematic data analysis [38]. - Adoption of Fletcher’s approach to identify recurring patterns (demi-regularities) at the CR real level [33]. | - Expansion of the initial codebook from 58 to 78 distinct codes (Multimedia Appendix 6). - Identification of three core themes (Figure S2): (1) centrality of clinical evidence, (2) importance of implementation considerations, and (3) absence of economic value consideration. |
| **2. Abduction** | - Implementation of Sætre and Van de Ven four-step abductive reasoning approach [41]: (1) observing puzzling facts in researchers’ decision-making patterns, (2) confirming these anomalies with empirical evidence, (3) formulating explanatory hypotheses, and (4) evaluating alternative explanations. | - Iterative analysis of empirical data and theoretical literature revealed two puzzling patterns in researchers' decision-making: (1) strong prioritization of clinical evidence while consistently deprioritizing economic considerations (despite acknowledging their importance), and (2) significant focus on implementation factors that actually align with economic value domains in HTA frameworks, despite researchers' claims of neglecting economic considerations. |
| **3. Retroduction** | - Investigation of causal mechanisms using Danermark's framework [44]. - Analysis of dependencies and interactions between the identified mechanisms, validated via three complementary triangulation methods (data, iterative, and investigators). - Visualization of complex relationships through QSD and CLDs. | - Recognition of three primary generative mechanisms: (1) the *Professional Norms*, (2) the *Researcher Experience*, and (3) the *Adoption Uncertainties*. - Developed CLDs for each mechanism (Figures 2-4), mapping the variables and potential causal links, which ultimately converged into an integrated single model (Figure 4) explaining the interactions between identified mechanisms and their influence on DTx decision-making. |
