# Supplementary material for: Understanding Economic Decision-Making in Digital Therapeutics Development: Qualitative Approach
Source: J Med Internet Res. 2025 Sep 16;27:e79746. doi: 10.2196/79746 (PMC12485261; doi:10.2196/79746)
Supplement: Multimedia Appendix 6 [file jmir_v27i1e79746_app6.docx]

| **Number** | **Code Category** | **Code** | **Frequency** | **Short description** | **Primary Sources** |
| --- | --- | --- | --- | --- | --- |
| 1 | Bias > | Anchoring | 7 | Anchoring to initial estimates, that is, researchers’ tendency to rely potentially too heavily on the first piece of information they encounter when making decisions. | DT > BDT based  CR > Cognitive constraints |
| 2 | Bias > | Availability heuristic | 2 | Related to the tendency to overestimate the likelihood of events based on how easily they come to mind. | DT > BDT based  CR > Cognitive constraints |
| 3 | Bias > | Confirmation bias | 7 | Researchers’ tendency to search for, interpret, and recall information in a way that confirms existing beliefs. | DT > BDT based  CR > Cognitive constraints |
| 4 | Bias > | Cultural | 1 | Influence of cultural background and values on research decisions and interpretations. | Code emerged from Chevannah’s (P14) transcript analysis |
| 5 | Bias > | Loss aversion | - | Related to the tendency to prefer avoiding losses over acquiring equivalent gains. | CT > BDT based  CR > Cognitive constraints |
| 6 | Bias > | Other | 7 | Miscellaneous cognitive biases affecting research decisions that do not fit into other specific categories. | Code emerged from Eisa’s (P4) transcript analysis |
| 7 | Bias > | Overconfidence | 14 | Related to the tendency to overestimate one's own abilities, knowledge, and accuracy of predictions. | DT > BDT based  CR > Cognitive constraints |
| 8 | Bias > | Professional training | 21 | Impact of professional education and experience on DTx development processes and related decisions. | Code emerged from Eva’s (P2) transcript analysis |
|  |  |  |  |  |  |
| 9 | Clinical validation > | Clinical value prioritization | 150 | Focus on generating evidence of DTx safety, efficacy, and health benefits. | Literature Review  CR > Actual Domain |
| 10 | Clinical validation > | Clinical trial results | 37 | Documented outcomes demonstrating DTx safety and efficacy from trials. | Literature Review  CR > Empirical Domain |
| 11 | Clinical validation > | Participant selection | 5 | Criteria for choosing appropriate study participants for clinical validation. | Literature Review  CR > Actual Domain |
| 12 | Clinical validation > | Study design decisions | 20 | Methodological choices for validation, including outcome measure selection. | Literature Review  DT > EUT based  CR > Actual Domain |
|  |  |  |  |  |  |
| 13 | Context > | Healthcare system characteristics | 10 | Structural aspects of healthcare systems affecting DTx implementation. | Code emerged from Cheah’s (P13) transcript analysis |
| 14 | Context > | Market fit | 42 | DTx's ability to address significant clinical needs in the target market. | Literature Review |
| 15 | Context > | Regulatory influence and considerations | 67 | Impact of potential (or the absence of) guidelines, standards, and requirements on DTx development. | Literature Review  CR > Real Domain |
| 16 | Context > | Reimbursement considerations | 42 | Planning for payment and coverage mechanisms. | Literature Review  DT > BDT based |
| 17 | Context > | Resource-related considerations | 102 | Constraints in funding, time, expertise, and other development resources (e.g., missing information or expertise needed for development Knowledge gaps). Defined as constraints on Agency, under CR. | Literature Review  DT > BDT based  CR > Cognitive constraints |
| 18 | Context > | Decision-making > | 28 | Environmental and situational factors influencing decision processes. | Code emerged from Ezra’s (P3) transcript analysis |
|  |  |  |  |  |  |
| 19 | DTx development > | Access | 7 | Considerations related to ensuring equitable access to the DTx solution across user populations. | Code emerged from Chevannah’s (P14) transcript analysis |
| 20 | DTx development > | Diversity of stakeholders | 104 | Involvement of varied stakeholders in the DTx development process. | Code emerged from Emma’s (P1) transcript analysis |
| 21 | DTx development > | Patient centricity | 45 | Focus on incorporating patient needs, preferences, and experiences throughout the DTx development process, ensuring the solution addresses real patient challenges and promotes engagement. | Code emerged from Emma’s (P1) transcript analysis |
| 22 | DTx development > | Patterns | 4 | Recurring approaches in development processes and decision-making. | CR > Empirical Domain |
| 23 | DTx development > | Process | 142 | Stages of technological innovation and development. | Code emerged from Emma’s (P1) transcript analysis |
|  |  |  |  |  |  |
| 24 | Eco. consideration > | Long-term costs | 10 | The long-term impact of DTx onto society. | Code emerged from Christoph’s (P12) transcript analysis |
| 25 | Eco. consideration > | Business model | 70 | Strategy for DTx monetization and market deployment. | Literature Review |
| 26 | Eco. consideration > | Clinical validation costs | 6 | Expenses for trials, recruitment, data collection, and analysis. | Literature Review |
| 27 | Eco. consideration > | Development costs | 14 | Expenses for initial creation and iterative improvements of the DTx. | Literature Review  DT > EUT based |
| 28 | Eco. consideration > | Direct medical and non-medical costs | 54 | Including costs of pharmaceutical treatment, of the DTx, health resource utilization, intervention-specific training, and participants’ time spent on the DTx. | Literature Review  DT > EUT based |
| 29 | Eco. consideration > | Eco. prioritization | 94 | Emphasis on economic considerations in development. | Code emerged from Eva’s (P2) transcript analysis |
| 30 | Eco. consideration > | Economic value awareness | 145 | Researchers’ understanding of the concept of economic value (e.g., strategies implemented to ensure economic viability of the DTx) | Literature Review |
| 31 | Eco. consideration > | Economic evaluation methods | 66 | Approaches for assessing DTx cost-effectiveness and value, such as cost-benefit analysis (CBA), cost-effectiveness analysis (CEA), or cost-utility analysis (CUA). | Literature Review  DT > EUT based > HTA |
| 32 | Eco. consideration > | Funding sources | 15 | Available financial resources for DTx development. | Literature Review  CR > Real Domain |
| 33 | Eco. consideration > | Implementation related | 114 | Awareness of practical deployment considerations, such as clinical workflow integration, provider training needs, or support requirements. | Literature Review  DT > EUT based |
| 34 | Eco. consideration > | Indirect medical and non-medical costs | 37 | Including costs related to the productivity impact and DTx maintenance. | Literature Review  DT > EUT based |
| 35 | Eco. consideration > | Influencing factors | 16 | Including participants’ baseline characteristics, reimbursement rate, treatment adherence, attrition rate, degree of clinical inertia and sustained DTx clinical effectiveness. | Literature Review |
|  |  |  |  |  |  |
| 36 | Emotional factors > | Confidence | 6 | Self-assurance in development decisions and approaches. | Code emerged from Surya’s (P10) transcript analysis |
| 37 | Emotional factors > | Fear and anxiety | 5 | Concerns, worries, or anxieties influencing decision-making processes. | DT > BDT based |
| 38 | Emotional factors > | Optimism and hope | 7 | Expressions of positive expectations or confidence influencing choices and strategic decisions. | DT > BDT based |
| 39 | Emotional factors > | Passion and commitment | 24 | Indications of personal investment, dedication, or emotional attachment which drive decision-making and persistence. | DT > BDT based |
| 40 | Emotional factors > | Pressure and stress | 11 | Mentions of time constraints, workload, or external demands affecting decision-making behavior. | DT > BDT based |
|  |  |  |  |  |  |
| 41 | Knowledge > | Best practices | 22 | Proven effective methods, techniques, and approaches in DTx development and implementation. | Code emerged from Eva’s (P2) transcript analysis |
| 42 | Knowledge > | Expert consultation processes | 26 | Methods for incorporating subject matter expertise. | Literature Review |
| 43 | Knowledge > | Feedback integration | 73 | Incorporation of stakeholder perspectives and input, such as patients and HCPs. | Literature Review  CR > Empirical Domain |
| 44 | Knowledge > | Market intelligence utilization | 13 | Use of market data in decision-making. | Literature Review |
| 45 | Knowledge > | Talent development | 4 | Strategies and processes for building and maintaining necessary expertise and skills within the development team, including training and knowledge transfer. | Code emerged from Cheah’s (P13) transcript analysis |
|  |  |  |  |  |  |
| 46 | Organizational context > | Institutional research priorities and mandates | 73 | Formal organizational goals directing research focus. | Literature Review |
| 47 | Organizational context > | Organizational culture toward commercialization | 48 | Institutional attitudes and practices toward research commercialization. | Literature Review |
| 48 | Organizational context > | Strategic alignment | 3 | Alignment between DTx goals and broader organizational objectives (e.g., level of leadership support). | Literature Review |
|  |  |  |  |  |  |
| 49 | Outcome evaluation > | Market potential evaluation | 19 | Assessment of commercial opportunities, market size and potential, and therefore of the DTx potential value. | Literature Review  DT > EUT based |
| 50 | Outcome evaluation > | Regulatory outcomes | 5 | Considerations related to obtaining regulatory approval in order to commercialize a DTx in specific markets. | DT > EUT based  CR > Empirical Domain |
|  |  |  |  |  |  |
| 51 | Persona > | Adoption related | 91 | Considerations (and concerns) related to DTx uptake by target users. | Code emerged from Surya’s (P10) transcript analysis |
| 52 | Persona > | Clinicians’ mindset | 45 | Unique perspectives and decision-making approaches that distinguish clinicians-researchers in their work. | Code emerged from Eva’s (P2) transcript analysis |
| 53 | Persona > | Engineer’s mindset | 34 | Unique perspectives and decision-making approaches that distinguish engineers-researchers in their work. | Code emerged from Eva’s (P2) transcript analysis |
| 54 | Persona > | Researcher’s mindset | 34 | Insights and perspectives that characterize researchers' general approach and thought processes. | Code emerged from Eva’s (P2) transcript analysis |
| 55 | Persona > | Researchers’ priorities | 108 | Day-to-day priorities of researchers, and therefore key considerations in decision-making processes. | Code emerged from Emma (P1) transcript analysis |
| 56 | Persona > | Researchers’ role | 95 | Interviewee's involvement and contributions across different stages of DTx development and technical lifecycle. It also captures the personal experiences of the researchers with DTx development. | Code emerged from Emma (P1) transcript analysis  CR > Empirical Domain |
|  |  |  |  |  |  |
| 57 | Probability > | Probability calculations | 10 | Objective and subjective probability calculations. | Literature Review  DT > EUT based |
| 58 | Probability > | Uncertainty | 13 | Acknowledgment of potential uncertainty. | Literature Review  DT > EUT based |
|  |  |  |  |  |  |
| 59 | Social influence > | Competitive pressure | 8 | Impact of market competition on decisions. | DT > BDT based |
| 60 | Social influence > | Norms | 30 | Established practices, standards, and unwritten rules that shape behavior within the DTx ecosystem. | DT > BDT based |
| 61 | Social influence > | Peer pressure | 3 | Direct and indirect influence exerted by professional peers and industry colleagues on decision-making. | DT > BDT based |
| 62 | Social influence > | Status and reputation concerns | 2 | Decision-making influenced by considerations of professional standing and organizational reputation. | DT > BDT based |
|  |  |  |  |  |  |
| 63 | Stakeholder relationships > | Communications | 17 | Related to the communications between the different stakeholder groups. | Code emerged from Shiva’s (P9) transcript analysis |
| 64 | Stakeholder relationships > | Cross-disciplinary collaboration structures | 72 | Formal and informal arrangements for working across different fields of expertise, encompassing collaborative networks that facilitate knowledge exchange. | Literature Review  CR > Actual Domain |
| 65 | Stakeholder relationships > | Group and power dynamics | 12 | Distribution and exercise of authority in decision-making (e.g., stakeholder alignment issues). It includes the “network effects”, i.e., the impact of existing relationships and connections on researchers’ decision-making and development choices. | Literature Review  CR > Actual Domain |
| 66 | Stakeholder relationships > | Industry-academia relationships | 21 | Connections and interactions between academic researchers and industry partners. | Literature Review  CR > Actual Domain |
| 67 | Stakeholder relationships > | PPP dynamics | 6 | Public-private partnership interactions and relationships. | Literature Review  CR > Actual Domain |
|  |  |  |  |  |  |
| 68 | Tech. development > | Features prioritization | 10 | Decisions about which features or capabilities to develop first. | Literature Review |
| 69 | Tech. development > | Interoperability considerations | 16 | Ability to integrate with other systems and platforms. | Literature Review |
| 70 | Tech. development > | Security and privacy features | 8 | Measures for protecting data and ensuring privacy. | Literature Review |
| 71 | Tech. development > | Tech. architecture | 5 | Choices and decisions about technological structure and components. | Literature Review |
| 72 | Tech. development > | Tech. limitations | 3 | Constraints imposed by technology or infrastructure. | Literature Review  CR > Real Domain |
| 73 | Tech. development > | Tech. prioritization | 67 | Focus on technological capability development. It could also be considered as a “pro-innovation bias”. | Code emerged from Emma’s (P1) transcript analysis |
| 74 | Tech. development > | User interface | 12 | Choices affecting how users interact with the DTx (e.g., decisions related to the end-user experience). | Literature Review |
|  |  |  |  |  |  |
| 75 | Utility > | Resource | 2 | Resource allocation-related decisions and considerations. | DT > EUT based  CR > Actual Domain |
| 76 | Utility > | Risk | 10 | Notions of risk and uncertainty evaluation. | DT > EUT based |
| 77 | Utility > | Scalability | 31 | Scalability potential of the future DTx intervention. | DT > EUT based |
| 78 | Utility > | Time | 1 | Time-value considerations (e.g., specific deadlines). | DT > EUT based |
