# Supplementary material for: Understanding Economic Decision-Making in Digital Therapeutics Development: Qualitative Approach
Source: J Med Internet Res. 2025 Sep 16;27:e79746. doi: 10.2196/79746 (PMC12485261; doi:10.2196/79746)
Supplement: Multimedia Appendix 7 [file jmir_v27i1e79746_app7.docx]

The first coding cycle employed descriptive and in vivo coding methods to identify first-order codes that preserved participants' original language and captured basic descriptive elements of the data. This process led to the development of new codes, while some existing codes were combined or refined to better reflect participants’ experiences. For example, “Knowledge > Patient Feedback Integration” was broadened to “Knowledge > Feedback Integration” to reflect participants' frequent reports of consulting physicians, nurses, and specialists. The notion of DTx adoption emerged early as a potentially important factor throughout the DTx process, leading to the creation of the “Implementation > Adoption” code. Through this iterative coding process, the framework expanded from 58 provisional codes to 78 distinct codes (Multimedia Appendix 6), demonstrating the iterative nature of the coding process [33]. Throughout this process, detailed notes for each interview transcript were maintained, documenting key discussion points, interpretations, potential participant quotes and changes to the codebook.

These first-order codes were then aggregated during the second coding cycle using pattern coding and focused coding techniques to develop more conceptual second-order categories that represented broader analytical groupings. For example, several first-order codes, such as “Economic value awareness,” “Business model,” and “Clinical validation costs” were synthesized into the second-order category, “Economic considerations.”

This progression from participant verbatim, through concrete first-order codes, to abstract themes followed Saldaña's principle of “code weaving”, where codes were integrated into a coherent analytical narrative that connected empirical observations with theoretical constructs (Figure S1) [38].

**Figure S1.** Verbatim to codes, subthemes, and themes.

**
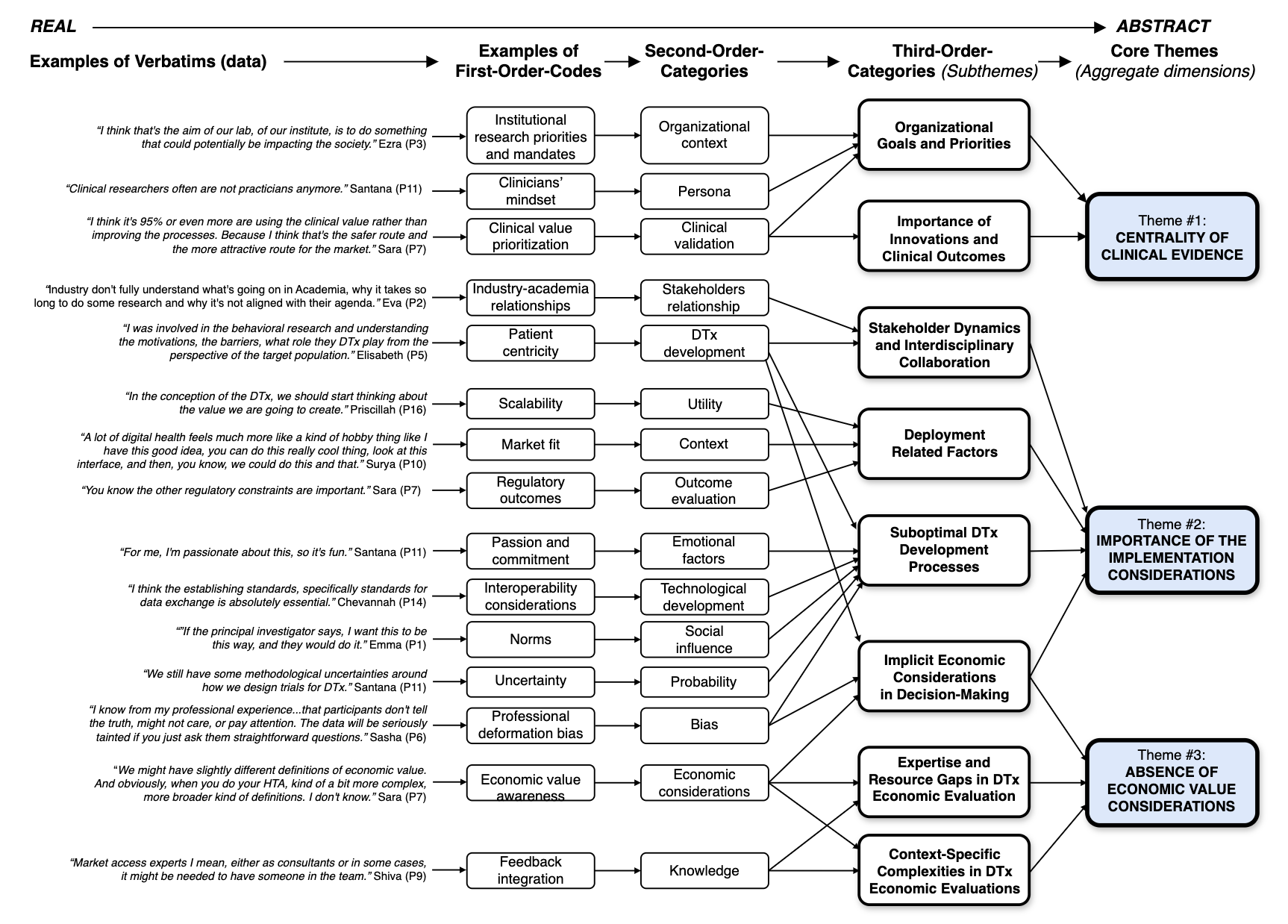
**
