# Supplementary material for: Understanding Economic Decision-Making in Digital Therapeutics Development: Qualitative Approach
Source: J Med Internet Res. 2025 Sep 16;27:e79746. doi: 10.2196/79746 (PMC12485261; doi:10.2196/79746)
Supplement: Multimedia Appendix 8 [file jmir_v27i1e79746_app8.docx]

| **Mechanisms** | **Representative Quotes** | **Context** | **Interpretation** |
| --- | --- | --- | --- |
| **Professional Norms** | “*As a researcher, my KPIs are focused on publications and clinical validation... economic considerations aren't part of our metrics.*” (Emma, P1)  “*The priorities would be focused on technical performance and the clinical value this solution can have*.” (Elisabeth, P5)  “*We were too focused on the clinical service delivery team [and did not engage some of the stakeholders'. So we developed that and then went through a couple of trials. One was a pre-test, post-test study first, now a randomized controlled trial to see whether it really works. At this point, we still have not done cost-benefit analysis. So it may be a money-losing intervention, we don't know, but we may be throwing money into the ocean. So this is one downside*.” (Camirah, P15) | Discussion of research priorities | Demonstrates how institutional structure and dynamics shape decision-making focus |
| **Researcher Experience** | “*From my engineering background, I naturally focus on technical performance... economic aspects weren't part of my training.*” (Ezra, P3)  “*I don't really consider myself an expert in this area at all. I have some knowledge, partly during training, there are specific components of our examinations that cover things like cost effectiveness.*” (Cheah, P13)  “*It's safer to follow guidelines. That's what we're trained to do throughout our training*.” (Surya, P10) | Reflection on professional training | Shows the influence of educational background on decision frameworks |
| **Adoption Uncertainties** | “*Implementation is always the biggest unknown... you can have perfect clinical results but fail completely in real-world adoption.*” (Christoph, P12)  “*The kind of the end goal is really about the adoptions, basically, the deployment and the adoptions of these technologies*.” (Priyah, P17)  “B*esides economics, it's the implementation part, like how do we assess or evaluate the fidelity or the adoption of this kind of program, how do we do stakeholder engagement, how to analyze their responses, how to overcome barriers and facilitators when you look at the big picture.*” (Camirah, P15) | Discussion of development challenges | Illustrates how adoption uncertainty shapes Dtx development-related decisions |
