# Supplementary material for: Understanding Economic Decision-Making in Digital Therapeutics Development: Qualitative Approach
Source: J Med Internet Res. 2025 Sep 16;27:e79746. doi: 10.2196/79746 (PMC12485261; doi:10.2196/79746)
Supplement: Multimedia Appendix 9 [file jmir_v27i1e79746_app9.docx]

To conceptualize these mechanisms and elucidate their interdependencies through a CLD, an iterative three-step process was implemented to: (1) identify influencing variables, (2) map relationships and assign polarity, and (3) validate and refine the model. Throughout each phase, three distinct categories of unit triangulation were incorporated [52], as shown in Figure S1 and detailed below.

**Figure S1.** Implementation of triangulation methodology for CLD construction in DTx decision-making.


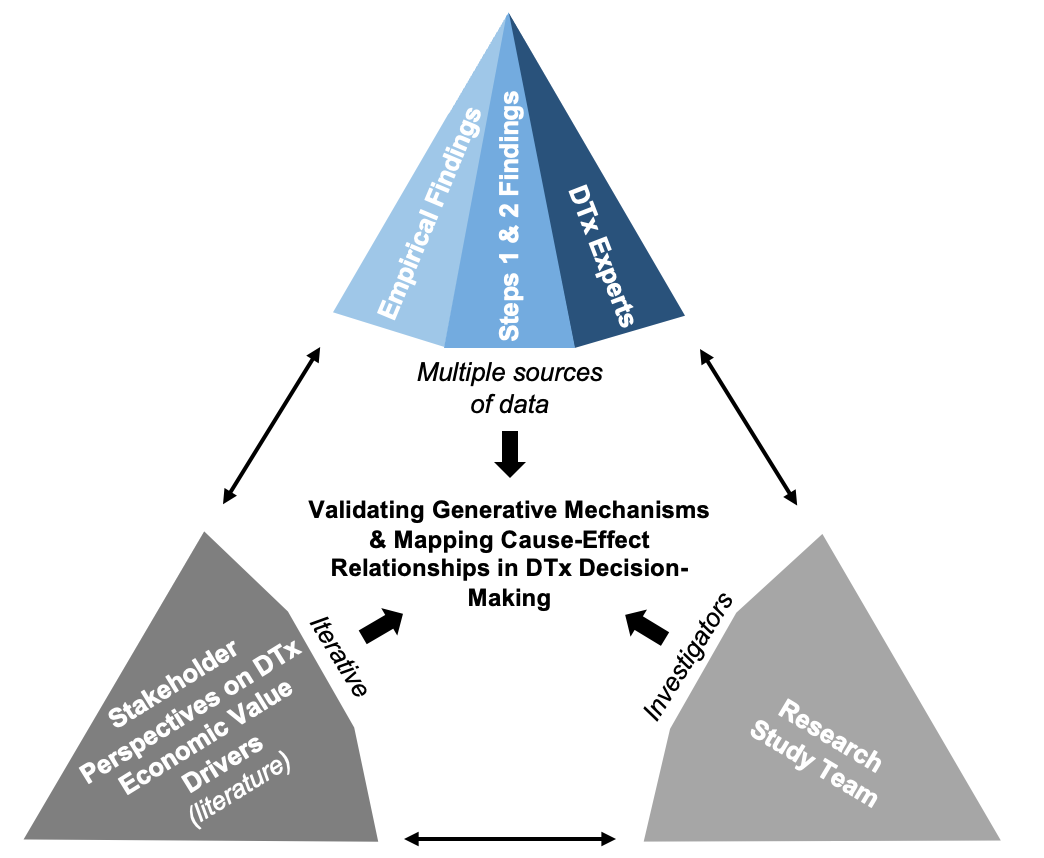


**Step 1: Variable identification**The data triangulation involved comparing **multiple sources of data**, including the study’s empirical findings from both steps 1 and 2, to identify potential variables and their relationships influencing researchers' decision-making processes. The extraction of variables and their relationships from the research data incorporated both explicit participant statements about causal relationships and pattern analysis across interviews to ensure comprehensive relationship mapping. Each potential relationship was systematically evaluated against three evidence criteria: (1) explicit mention in participant interviews, (2) support in existing literature, and (3) logical consistency with decision-making theory. Relationships meeting at least two criteria were included in the model.

**Step 2: Relationship mapping and polarity assignment**In parallel with variable identification, **iterative triangulation** was employed through systematic comparisons with literature findings, including the authors’ previous work, which had established both variables and causal relationships among eight clusters of factors influencing DTx economic value [22]. This methodological approach enabled critical comparative analysis between existing literature and refined conceptualization of variables influencing researchers' decision-making processes, and their interdependencies, leading to a CLD for each of the three generative mechanisms (Figures 2-4).

Relationship polarities (positive or negative) were established by analyzing directional influence between variables, supported by literature citations and participant quotes. Positive relationships indicate that variables move in the same direction—both increasing or both decreasing together. Negative relationships indicate opposite movements. Relationship strength (strong or weak) was determined by analyzing the frequency and emphasis of these connections within the data.

Feedback loops were identified through manual loop tracing—specifically by tracing paths through the causal network diagram and identifying where variables connect back to themselves through a series of relationships. Common system archetypes that appear frequently across different domains were also sought, such as “limits to growth” or “fixes that fail,” with consideration given to the characteristics of their feedback structures [58,59,91]. Each loop was categorized as either reinforcing (R) or balancing (B) based on the product of polarities along the loop. Reinforcing loops amplify change in a consistent direction, creating exponential growth or decline patterns, while balancing loops counteract change and tend toward stability or equilibrium. Each loop was named according to its primary function within the system, with documentation of its constituent variables and potential impact on system behavior.

**Step 3: Model validation and refinement**To validate and refine the model, as new versions of the CLDs emerged, they were discussed with the research study team through **investigator triangulation** to analyze and interpret findings, including reviewing codes when needed to ensure convergence. As part of the iterative process, discussions with three DTx experts who met the study inclusion criteria but were outside the original sample were scheduled at different time intervals to interpret, validate, and refine emerging versions of the individual mechanisms CLD (Figures 2-4), which over time converged into a single integrated refined CLD (Figure 4), illustrating the generative mechanisms and their manifestations shaping economic considerations in DTx development.
