# Supplementary material for: Understanding Economic Decision-Making in Digital Therapeutics Development: Qualitative Approach
Source: J Med Internet Res. 2025 Sep 16;27:e79746. doi: 10.2196/79746 (PMC12485261; doi:10.2196/79746)
Supplement: Multimedia Appendix 10 [file jmir_v27i1e79746_app10.docx]

This appendix summarizes the results from step 1 (Coding and identification of demi-regularities) and step 2 (Abductive reasoning process), as outlined in the data analysis methodology summary (Multimedia Appendix 5). These results informed the generative mechanisms retroduced in step 3, which are detailed in the Results section of the main paper.

**Emerging core themes (step 1)**First, the centrality of clinical evidence emerged as the most prominent demi-regularity, quantitatively corroborated by the highest coding frequency for the “Clinical Validation > Clinical value prioritization” code across the transcript corpus. Despite EUT's prediction that researchers would systematically evaluate all value components, participants consistently steered discussions toward clinical considerations. The predominance of clinical considerations became particularly evident through researchers' implicit interpretations of key terminology. When participants employed terms, such as “validation” and “generating evidence,” these consistently referenced clinical trials and clinical evidence generation rather than other forms of validation (technical or economic).

This clinically driven perspective extended to researchers' conceptualization of “value” of a DTx, which they predominantly viewed through the lens of clinical needs and safety/efficacy evidence. This pattern, which emerged consistently across clinician-researchers, was exhibited by most health systems and social science researchers and was frequently observed by practitioner-researchers. Notably, interviews with research engineers revealed a more nuanced pattern. Within this group, technological “performance” of the DTx, and what one participant termed “shiny engineering innovations” emerged as equally significant factors in their decision-making frameworks. This variation suggests a potential disciplinary influence on value assessment approaches within DTx research and development.

Second, implementation factors played a crucial role in development decisions, revealing tensions between rational planning and behavioral responses to uncertainty. Participants emphasized factors like “manpower utilization”, “workflow integration,” “care pathway changes”, clinical team “productivity” impacts, and “acceptability” from both patients and care teams. As Cheah (P13) articulated, “*when you implement a [DTx] solution in a clinical setup, most likely it's not going to work out. Implementation is an issue, such as adherence and adoption of a technology*”. Emma (P1) elaborated on this perspective: “*There's obviously the medical value of the DTx, but it's also the workflow fit, and the human factors more broadly considered. What is the patient journey, where the solution is going to fit, understand the physician's decision making, their involvement, are the nurses involved?*” Uncertainties surrounding implementation and adoption may foster risk-averse decision-making, potentially limiting innovation in traditionally conservative healthcare settings. Elisabeth (P5) emphasized the importance of balancing internal constraints to avoid “*overburdening some of the early thinking point*” and maintaining an innovative culture. This risk-aware approach could also drive more rigorous pre-implementation planning during early DTx development, including new frameworks for risk assessment and controlled experiments. The study findings revealed that researchers typically employ multiple validation approaches to address implementation uncertainties, including physician and patient consultations to comprehensively evaluate needs and perspectives as well as pilot studies and usability testing.

Third, the absence of economic value considerations in early development stages emerged as a consistent pattern, reflecting the BDT's concept of bounded rationality. With a single exception (Cheah, P13), both clinician-researchers and research-engineers consistently indicated that economic factors fell outside their priorities or perceived responsibilities. Researchers also expressed uncertainty about incorporating economic considerations into their DTx development processes. Cheah (P13) stood as the sole exception, noting: “*In our ongoing study, the cost-effectiveness of our model of care is the primary endpoint, and the secondary endpoints are actually the efficacy and safety to supplement prior evidence we have on those aspects*.” This systematic de-prioritization of economic considerations in the sample deviates from the EUT's prediction of comprehensive utility maximization, creating potential blind spots in decision-making.

These demi-regularities suggest a complex interplay between rational decision-making frameworks and behavioral influences, with interesting tensions between EUT predictions and BDT observations, shaping how researchers approach DTx development decisions.

**Puzzling facts and plausible explanations (step 2)**

The second step, following Sætre and Van de Ven four-step abductive reasoning approach [41], identified puzzling facts and provided plausible explanations for them. As discussed above, a key empirical finding was researchers' predominant focus on clinical evidence, either as a single priority or combined with technological performance considerations in DTx development. Surprisingly, researchers focused almost exclusively on clinical benefits, leaving little room for other forms of value evidence, particularly economic considerations or process-oriented categories (e.g., DTx that enhance self-management capabilities, support informed decision-making, or reduce healthcare access barriers). This finding contrasts with researchers' acknowledged importance of such factors for successful implementation [22], as well as with healthcare systems' evolution since 2006—from the “triple aim” of improving care, health, and cost to the “Quadruple Aim” framework that adds meaning at work [92]. Modern healthcare frameworks recognize that, given limited resources, the economic aspects of healthcare delivery cannot be separated from clinical considerations. Germany's Digital Health Applications (DiGA) framework exemplifies this integrated approach, legitimizing “*patient-relevant improvements in structure and process*” alongside direct medical benefits when evaluating DTx for market approval [93].

The dominance of clinical evidence permits multiple theoretical interpretations. At its core, it likely reflects a deeply embedded institutional logic within healthcare, where clinical outcomes have historically served as the foundation of medical decision-making. Organizational structures and research priorities—whether oriented toward pure or translational research—may also influence researchers’ decision-making patterns.

Participants viewed economic considerations as outside their domain of expertise, expressing uncertainty regarding economic evaluations and their inherent complexity for DTx. This represents a form of constrained utility maximization where researchers optimize within familiar domains rather than across all value dimensions (clinical, technical, and economic). This selective optimization contrasts with EUT's prediction of comprehensive value assessment and maximization but aligns with BDT's concept of bounded rationality. While researchers wanted “their” DTx to be of use, their tendency toward “familiarity” was observed —defaulting to clinical metrics rather than pursuing comprehensive value assessments—and “satisficing behavior,” choosing the first option that meets minimum acceptability levels. As articulated by Camirah (P15): “*We tried doing some form of costing analysis, but we are not sure whether we are holistic enough in presenting the project costings to higher management. So that's something we are still struggling with. We may be throwing money into the ocean…*”

The systematic deprioritization, or absence, of economic considerations throughout DTx development, may be attributed to a lack of awareness of their importance at individual and/or institutional levels, especially in later stages of the DTx process, for implementation, adoption and for potential reimbursement decision. It can also be hypothesized that researchers were led back to more familiar domains by a certain level of economic literacy limitations in the interview sample. Moreover, most researchers conceptualized the DTx process as linear and chronological or sequential, with a stepwise approach. This sequential decision-making framework may explain the deferral of economic considerations to later stages, further supporting the use of heuristic decision-making strategies outlined in the BDT framework.

Researchers consistently reported uncertainty surrounding DTx implementation and adoption as a potential “barrier” or “complexity,” which led to the second major theme discussed above. Their acknowledgment of these aspects as significant threats to DTx success was surprising (to the research study team). Although researchers reported that economic elements did not enter their decision-making process, many of the implementation-related aspects they did consider correspond to direct and indirect medical and non-medical costs associated with DTx in HTA terminology. Indeed, the pattern of implementation prioritization aligns with important domains in HTA frameworks [17,94,95]. Although researchers reported limited consideration of economic factors throughout DTx development, the findings suggest, therefore, that certain aspects of DTx economic value may be more thoroughly integrated throughout DTx development than either articulated by the researchers or previously evidenced [22].

In conclusion, the abductive analysis revealed key puzzling facts in DTx researchers' decision-making processes, suggesting a complex interplay between institutional logics, individual expertise boundaries, and conceptual frameworks. This abductive step provided plausible explanations for these phenomena, which are further examined in the retroductive step (step 3) of the analysis (see Results section) to develop a deeper understanding of the underlying mechanisms and structures influencing DTx researchers' decision-making patterns.
