# Supplementary material for: Understanding Economic Decision-Making in Digital Therapeutics Development: Qualitative Approach
Source: J Med Internet Res. 2025 Sep 16;27:e79746. doi: 10.2196/79746 (PMC12485261; doi:10.2196/79746)
Supplement: Multimedia Appendix 11 [file jmir_v27i1e79746_app11.docx]

| **Primary within-mechanism loops** | |
| --- | --- |
| R1. Clinical Value Loop  **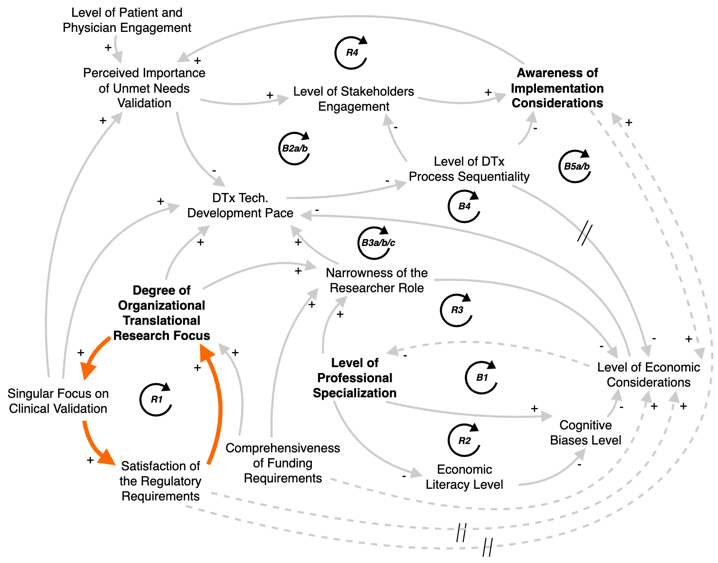** | R2. Literacy Loop  **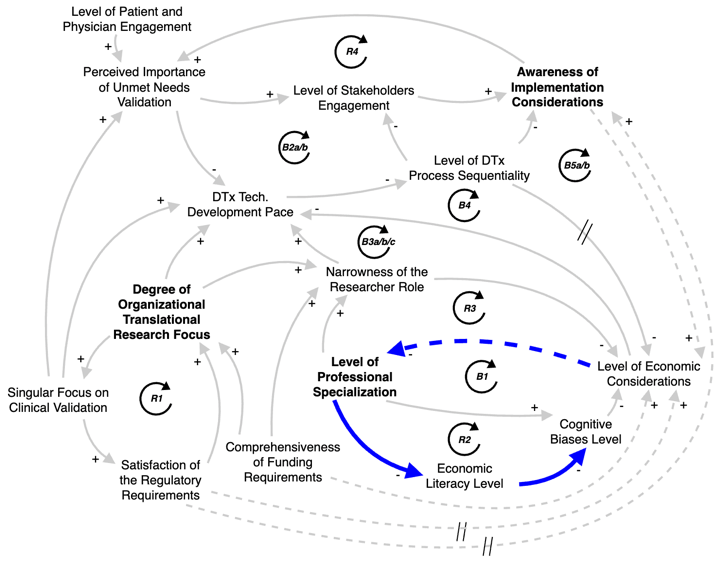** |
| B1. Bias Loop  **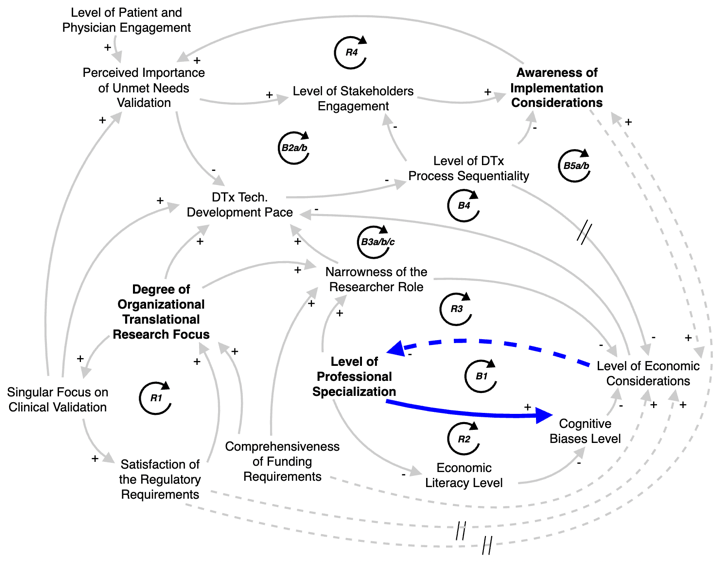** | R3. Role Boundary Loop  **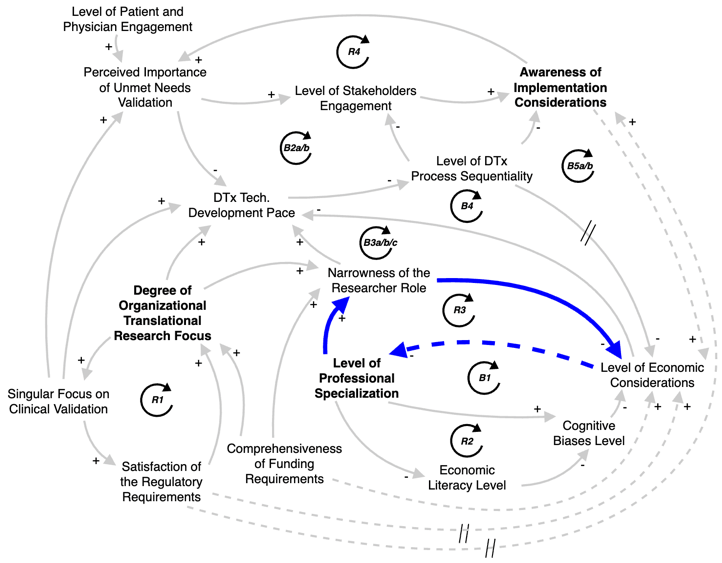** |

| B2a. Implementation-Driven Development Loop  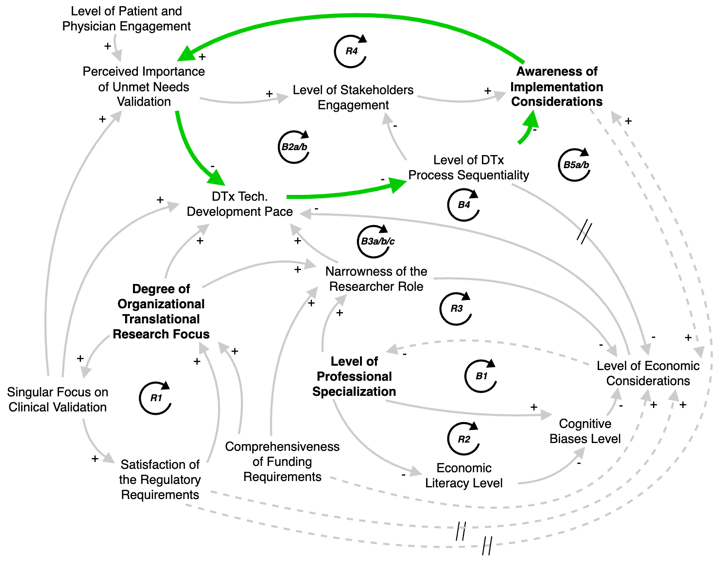 | B2b. Stakeholder-Driven Development Loop  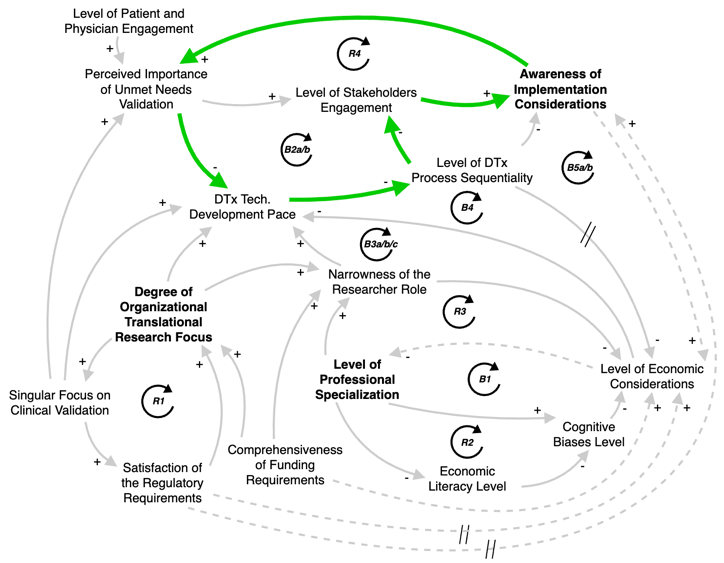 |
| --- | --- |
| R4. Collaboration Loop  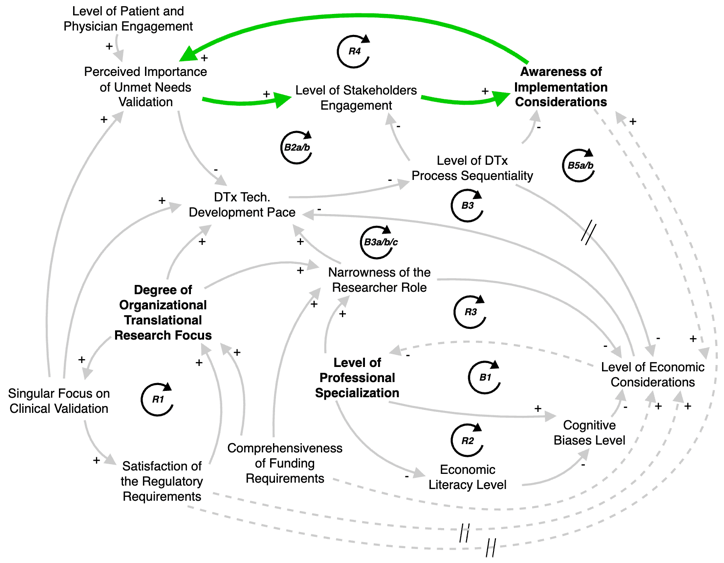 |  |

| **Secondary cross-mechanism loops** | |
| --- | --- |
| B3a. Specialization-Technology Loop  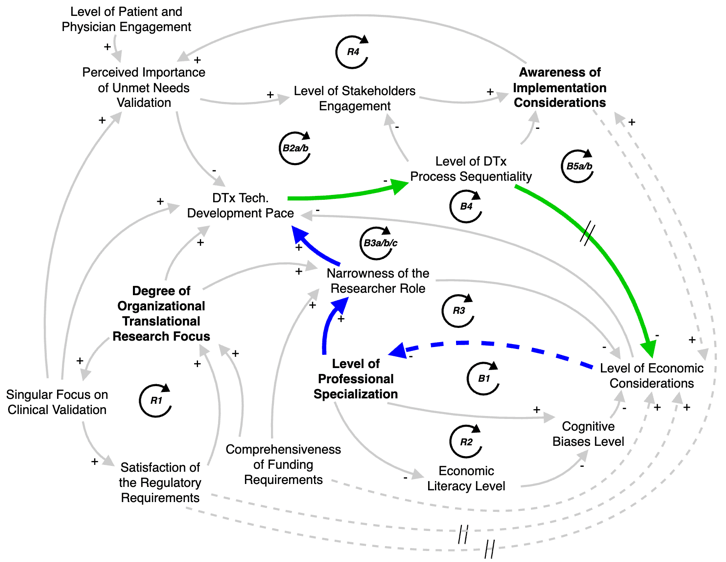 | B3b. Implementation-to-Specialization Loop  **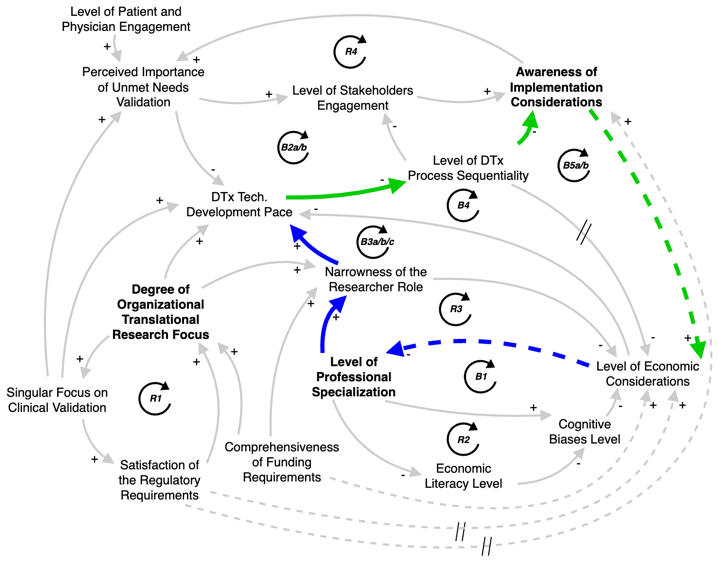** |
| B3c. Stakeholder-Guided Implementation-to-Specialization Loop  **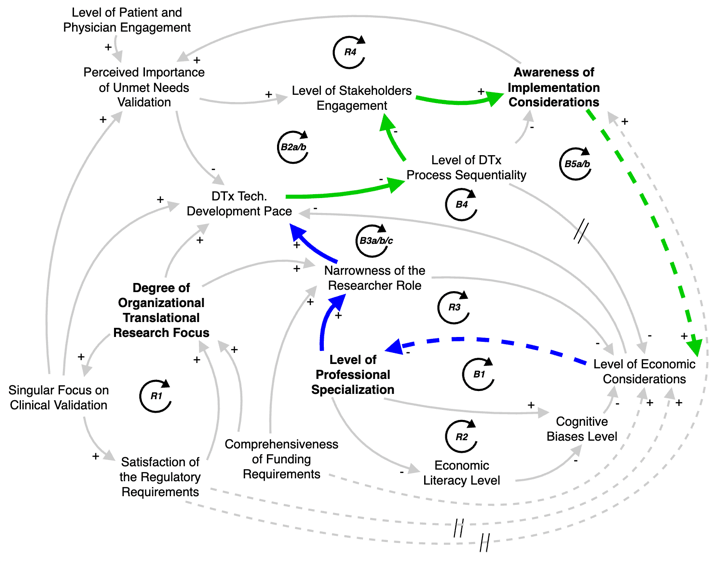** | B4. Development Efficiency Loop  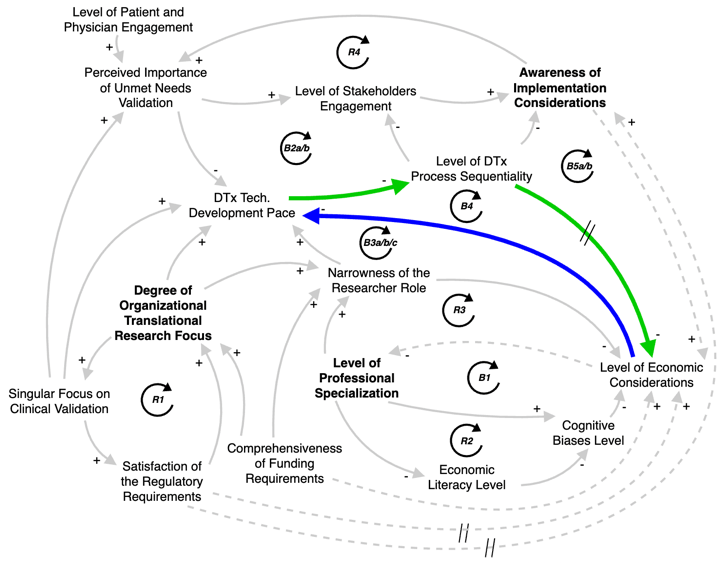 |

| B5a. Implementation-Economic Development Loop  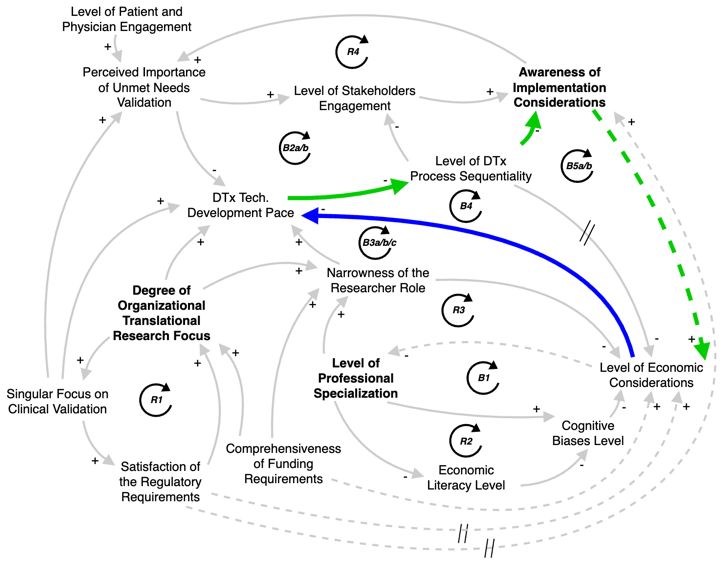 | B5b. Stakeholder-Informed Implementation-Economic Loop  **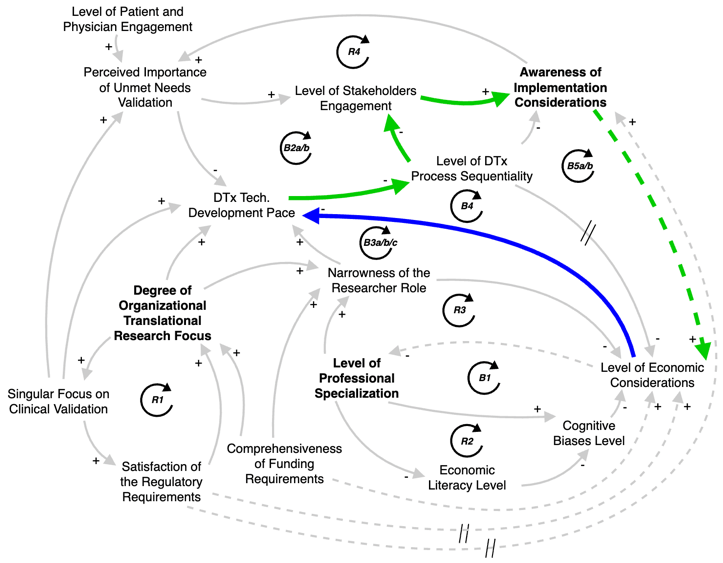** |
| --- | --- |
