# Supplementary figures and images for: Understanding Economic Decision-Making in Digital Therapeutics Development: Qualitative Approach
Source: J Med Internet Res. 2025 Sep 16;27:e79746. doi: 10.2196/79746 (PMC12485261; doi:10.2196/79746)

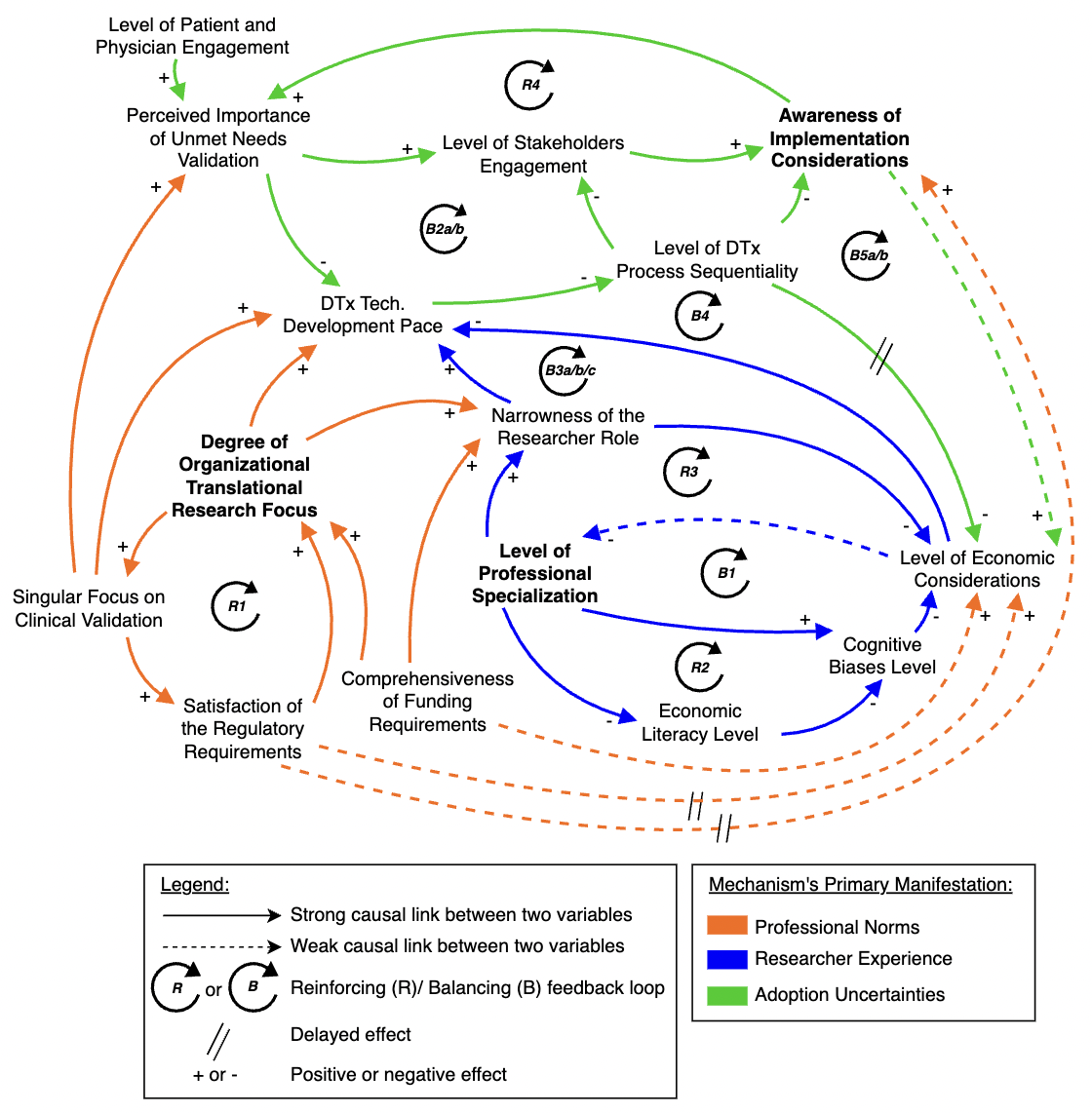

Supplement: Multimedia Appendix 12 [file jmir_v27i1e79746_app12.docx]
